# Deficiency of Antioxidative Paraoxonase 2 (Pon2) Leads to Increased Number of Phenotypic LT-HSCs and Disturbed Erythropoiesis

**DOI:** 10.1155/2021/3917028

**Published:** 2021-06-25

**Authors:** Lisa Spiecker, Ines Witte, Julia Mehlig, Viral Shah, Markus Meyerhöfer, Patricia S. Haehnel, Victoria Petermann, Andrea Schüler, Piyush More, Nina Cabezas-Wallscheid, Sven Horke, Andrea Pautz, Andreas Daiber, Daniel Sasca, Thomas Kindler, Hartmut Kleinert

**Affiliations:** ^1^Department of Pharmacology, Johannes Gutenberg University Medical Center, Mainz, Germany; ^2^Department of Hematology, Medical Oncology and Pneumology, University Medical Center, Mainz, Germany; ^3^University Cancer Center of Mainz, Germany; ^4^Max Planck Institute of Immunobiology and Epigenetics, 79108 Freiburg, Germany; ^5^Department of Cardiology, Cardiology I, Johannes Gutenberg University Medical Center Mainz, Germany

## Abstract

**Background:**

Long-term hematopoietic stem cells (LT-HSCs) reside in bone marrow niches with tightly controlled reactive oxygen species (ROS) levels. ROS increase results into LT-HSC differentiation and stem cell exhaustion. Paraoxonase 2 (PON2) has been shown to be important for ROS control.

**Objectives:**

We investigate the effects of inactivation of the *PON2* gene on hematopoietic cell differentiation and activity.

**Methods and Results:**

In young mice with inactivated *Pon2* gene (*Pon2*^−/−^, <3 months), we observed an increase of LT-HSCs and a reduced frequency of progenitor cells. In competitive transplantations, young *Pon2^−/−^* BM outcompeted WT BM at early time points. ROS levels were significantly increased in *Pon2^−/−^* whole BM, but not in *Pon2^−/−^* LT-HSCs. In more differentiated stages of hematopoiesis, *Pon2* deficiency led to a misbalanced erythropoiesis both in physiologic and stress conditions. In older mice (>9 months), *Pon2* depletion caused an increase in LT-HSCs as well as increased levels of granulocyte/macrophage progenitors (GMPs) and myeloid skewing, indicating a premature aging phenotype. No significant changes in ROS levels in old *Pon2^−/−^* LT- and short-term (ST-) HSCs were observed, but a significant reduction of spontaneous apoptotic cell death was measured. RNA-seq analysis in *Pon2*^−/−^ LT-HSCs identified overrepresentation of genes involved in the C-X-C chemokine receptor type 4 (Cxcr4) signaling, suggesting compensatory mechanisms to overcome ROS-mediated accelerated aging in hematopoietic progenitor cells.

**Conclusions:**

In summary, our current data indicate that PON2 is involved in the regulation of HSC functions.

## 1. Introduction

Aerobic metabolism is inevitably linked to the production of reactive oxygen species (ROS) such as superoxide, hydrogen peroxide, and hydroxyl radicals, which may have harmful effects on normal cellular function [1]. A tight balance between generation and detoxification of ROS has been shown to modulate cell physiology and development through redox signaling (low concentrations of ROS acting as signal molecules in physiologic processes) [2] and oxidative stress (high concentration of ROS exceeding the detoxification ability of cells). Oxidative stress results in destruction of proteins, DNA, and membrane lipids [3] and has been described to be involved in carcinogenesis [4], cardiovascular diseases [5], and aging [6].

The family of paraoxonase (PON) enzymes consists of three proteins PON1, PON2, and PON3 that differ in their enzymatic activity, localization, and regulation [7]. PON2 is a ubiquitously expressed protein located exclusively intracellularly [8]. PON2 exerts antioxidative and anti-inflammatory functions and displays important effects in diseases dominated by oxidative stress [9]. PON2 modulates mitochondrial function and reduces the release of superoxide from the inner mitochondrial membrane [10]. PON2 also displays a protective effect against lipid peroxidation [11] and intracellular ROS formation [12]. Our group showed recently that dysregulated redox regulation in mice with inactivated *Pon2* gene (*Pon2^−/−^*) causes endothelial dysfunction, vascular inflammation, and tissue factor-dependent hypercoagulability [13]. As a result of its antioxidative activity, antiapoptotic functions of PON2 have also been described both in mitochondria-related [14] and ER stress-related [12] apoptosis.

Hematopoiesis describes the hierarchically coordinated production of all blood cells with hematopoietic stem cells (HSCs) sitting at the apex. HSCs are characterized by their lifelong self-renewal ability and their capability to differentiate into all lineage committed progenitor cells [1]. To maintain hematopoiesis, the tight balance between differentiation and self-renewal in HSCs must be strictly regulated [15]. Defects in this balance lead to hematopoietic insufficiency and/or to the development of hematopoietic malignancies. In adult organisms, most HSCs are located in the bone marrow (BM). Different cell types, soluble factors, and anatomical structures collaborate to maintain HSC function. This delicate environment is referred as “niche” [16]. The BM niche and in particular the endosteal niche are characterized by a low oxygen concentration. A more restricted access to oxygen is likely to result in lower ROS levels. Analyses have shown that ROS are important to regulate the balance between self-renewal and differentiation of stem cells. Low levels of ROS are important to maintain the multipotency of these cells, whereas higher ROS levels would commit them to a restricted lineage [17, 18]. The low oxygen tension in the niche supports the ability of HSCs to self-renew and to stay quiescent. Self-renewing HSCs use anaerobic glycolysis as the main energy source to adapt to hypoxic conditions and meet the relative low energy needs of HSC [19]. However, the mitochondrial oxidative phosphorylation program is used if HSCs start to proliferate and differentiate [20]. Therefore, the primitive multipotent quiescent long-term (LT-) HSC is located in the endosteal niche [21]. In addition, ROS levels are tightly regulated by intrinsic mechanisms, e.g., regulation via the transcription factors FoxO1-3 [22].

The ability of stem cells to regenerate cells or tissues declines with age [23]. Compared to young animals, HSCs from aged animals display defined differences such as functional changes in homing and differentiation [24], enhanced ROS production, inflammation, and apoptosis [25, 26]. In older organisms, hematopoiesis displays a preferential generation of myeloid cells on the expense of lymphoid cells. This so-called myeloid skewing and the related immunosenescence seem to result from the clonal expansion of myeloid-committed hematopoietic stem and progenitor cells (HSPCs) and the reduction of lymphoid-committed HSPCs [24, 26].

The process of erythroid commitment and differentiation—termed as “erythropoiesis”—represents another crucial “checkpoint” of ROS-dependent regulation [27]. Erythropoiesis results in the production of red blood cells (RBCs) [28] from megakaryocyte/erythrocyte precursor cells (MEPs) [28]. Erythroid precursors are exposed to some of the highest ROS levels; however, similar to LT-HSCs, they also possess a large number of defense mechanisms against ROS and other insults [29]. The importance of ROS in erythroid maturation is supported for instance by the abnormalities of hematologic parameters in genetic diseases that lead to deficiency of mechanisms involved in antioxidation defence/reduction [30–32].

As mentioned above, HSC are mostly quiescent, show low metabolic activity with dependence on anaerobic glycolysis, and are prone to stimulation and damage by oxidative stress. PON2 is an antioxidant and antiapoptotic enzyme. Besides its important effects in the cardiovascular system, the antioxidative/antiapoptotic effects of PON2 seem to be exploited by different tumor cell types to enhance growth and resistance to chemotherapy [33]. Although PON2 expression has been correlated with the pathology of different forms of leukemia [33], the role of PON2 in hematopoiesis has not been analyzed. Therefore, the current study was performed to analyze the general involvement of PON2 in hematopoiesis.

## 2. Materials and Methods

### 2.1. Materials

All cell culture grade plastic materials were obtained from Greiner Bio-One, Frickenhausen, Germany, or SARSTEDT, Nümbrecht, Germany. All chemicals (as not otherwise stated), fetal calf serum, IgG from rat serum, RPMI 1640, Dulbecco's phosphate-buffered saline (PBS), Proteinase K, and Taq Polymerase were from Sigma, Deisenhofen, Germany. CM-H_2_DCF-DA was obtained from Molecular Probes/Thermo Fisher Scientific, Dreieich, Germany. L-012 (8-amino-5-chloro-7-phenyl-pyrido [3,4-d]pyridazine-1,4(2H,3H)dione) was obtained from Wako Chemicals, Richmond, U.S.A. The penicillin/streptomycin solution (100x; 10.000 U/ml penicillin, 10.000 *μ*g/ml streptomycin), Dulbecco's modified Eagle medium (DMEM), and GlutaMax™ were obtained from Gibco/Thermo Fisher Scientific, Dreieich, Germany. The High-Capacity cDNA Reverse Transcription Kit and the Arcturus® PicoPure® RNA Isolation Kit were purchased from Applied Biosystems, Darmstadt, Germany. The peqGOLD Total RNA Kit, peqGOLD TriFast™, and the dNTP-Mix were purchased from Peqlab, Darmstadt, Germany. The PrecisionPLUS 2x qPCR MasterMix with SYBR green was obtained from Primer Design, Chandler's Ford, United Kingdom. The Anti-Rat/Hamster Ig, *κ*/Negative Control (FBS∗) Compensation Particles Set, BD Cytofix/Cytoperm™ Fixation/Permeabilization Kit, BD™ CompBeads, PE Annexin V Apoptosis Detection Kit I, anti-Ki-67 antibody, and Perm/Wash buffer were obtained from BD Biosciences, Heidelberg, Germany.

### 2.2. Cell Culture

Murine hematopoietic precursor cell-7 (HPC-7 [34]) and BA/F3 pro B cells [35] were cultured as previously described [36]. To analyze the effect of the ROS generator 2,3-dimethoxy-1,4-naphthalenedione (DMNQ) [37], the cells were plated in 6-well plates and treated with 10 *μ*M DMNQ (solved in DMSO) or DMSO (control) for 2 to 8 h.

### 2.3. Mice and Approval of Animal Studies

PON2-deficient mice were generated by insertion of a loxP-site flanked, *β*-geo-containing gene trap vector into *Pon2* intron 2 [38]. Consequently, Pon2 protein expression is reduced by about 95%. These *Pon*2-deficient mice are referred to as *Pon2*^−/−^ mice. Wild-type (WT), C57BL/6J, C57BL/6-Ly5.1, and *Pon2*^−/−^ mice were housed in the translational animal research center of the JGU Mainz. All strains had access to water and standard chow diet ad libitum. Experimental mice were 10-14 weeks old when called “young” or more than 9 months old when called “aged.” The animals were sacrificed by i.p. injection of 2% pentobarbital (0.4 ml/25 g body weight). All animal studies were approved by the Ethical Committee and Landesuntersuchungsamt Rheinland-Pfalz (#23177-07/G13-1-055).

### 2.4. Blood Drawing and Analysis

After injecting a lethal dose of pentobarbital intraperitoneally (i.p.), intracardial blood was obtained for later analysis on the Sysmex XP Hematology Analyzer or HEMAVET using a syringe coated with citrate solution (Sigma) and a 26 G needle.

For smaller amounts of blood at multiple points in time, e.g., for analyzing erythrocyte turnover and stress erythropoiesis, mice were gently restrained, while blood was drawn by scratching the *Vena caudalis mediana* and immediately transferred to an EDTA-coated reaction vessel.

### 2.5. In Vivo Biotinylation for Analysis of the Erythroid Lifespan/Turnover

The erythroid lifespan/turnover was analyzed using biotin labeling according to published protocols [39, 40].

For the erythroid cell biotinylation *in vivo*, WT and Pon2^−/−^ mice were injected into the tail vein (i.v.) using a 26 G ½ cannula with 100 *μ*l of a sulfo-NHS-LC-biotin solution (30 mg/ml), resulting in a labeling rate of 80-95% of the circulating erythrocytes. The first blood sample was taken after 30 minutes in order to determine the individual starting value of the biotinylation of each mouse. Subsequent blood samples were taken daily (first 5 days) and then at intervals of 5 days, from day 20 at intervals of 7 days. Approximately 10 *μ*l blood was drawn and PBS+2% FCS as well as streptavidin APC-Cy7 (1 : 250) and Ter119 APC (1 : 500) (both from eBioscience Thermo Fisher Scientific) was added. After incubating for 15 minutes in the dark and on ice, samples were washed and analyzed on the FACSCanto™ II flow cytometer.

### 2.6. Induction of Hemolytic Anemia by Phenylhydrazine

Approximately 80 *μ*l blood was drawn from WT and Pon2^−/−^ mice by scratching the *Vena caudalis mediana*, transferred to an EDTA-coated reaction vessel, and examined using the Sysmex XP Hematology Analyzer. The mice were then injected with 50 mg/kg phenylhydrazine hydrochloride (Sigma-Aldrich) or PBS (for control animals) i.p. Injection was performed at days 1 and 3.

### 2.7. Flow Cytometry and Cell Sorting

Cell suspensions from the liver were obtained by pushing the organ through a 100 *μ*m cell strainer. Single-cell suspensions from BM were obtained by flushing tibial and femoral bones using RPMI/2% FCS and subsequently filtering the suspension through a cell strainer cap. The targets for the antibodies (all from eBioscience Thermo Fisher Scientific, Dreieich, Germany, unless stated otherwise) used for staining of differentiated BMCs were B220 (CD45R) (APC); CD3e (PE); CD4 (APC); CD8 (Ly2) (PE); ckit (PerCP-eFluor710); CD11b (APC); Gr1 (Ly6G/C) (PE); CD19 (PE, BioLegend, San Diego, U.S.A.); Ter119 (APC); CD71 (PE); and CD138 (Brilliant violet 421, BD Biosciences). For identification of hematopoietic stem and progenitor cells (LT-HSCs, ST-HSCs, MPPs, CMPs, GMPs, and MEPs), cells were incubated with a lineage cocktail of biotin-conjugated antibodies directed against CD3, CD4, CD5, CD8 (Ly2), CD11b (only LT-HSCs, ST-HSCs, and MPPs), CD127 (only CMPs, GMPs, and MEPs; BioLegend), B220 (CD45R), Gr1 (Ly6G/C), and Ter119. After washing, cells were incubated with streptavidin (APC-Cy7), Sca-1 (PECy7), ckit (APC), CD135 (PE, only LT-HSCs, ST-HSCs, and MPPs, BioLegend), CD150 (Alexa Fluor 488, only LT-HSCs, ST-HSCs, and MPPs, BioLegend), CD16/32 (PE, only CMPs, GMPs, and MEPs, BD), and CD34 (Alexa Fluor 488, only CMPs, GMPs, and MEPs, BD). For quantification of apoptotic HSCs, CD135 (PE) was replaced by annexin V (PE), and for measurement of total ROS via H_2_DCF-DA staining, CD150 (Alexa Fluor 488) was replaced by CD150 (Brill. violet 421). Data acquisition was done with a FACSCanto II (BD Biosciences) and analyzed using BD FACSDiva™ Software.

For sorting, HSPCs were enriched using EasyStep™ Mouse Hematopoietic Progenitor Cell Isolation Kit (StemCell Technologies, Cologne, Germany), stained with the antibodies mentioned before, and sorted on a FACSARIA™ II SORP Flow Cytometer Cell Sorter (BD Biosciences). All gating strategies are shown in Figure S4.

### 2.8. Reciprocal Bone Marrow (BM) Transplantation

BM cells (BMCs) of WT or *Pon2*^−/−^ donor mice were isolated by flushing tibial and femoral bones using RPMI/2% FCS/1% penicillin-streptomycin followed by BM cell resuspension in DMEM. By injection of 5 × 10^6^ BMCs intravenously into recipient mice of the respective genotype 24 hours after lethal irradiation (Cs137, one dose of 9 Gy; this radiation dosage was confirmed to be lethal after 8-10 days), *Pon2^−/−^* and WT BM chimeras were generated. BM-transplanted mice were analyzed after confirmation of blood cell *Pon2* mRNA expression by qRT-PCR, no earlier than 21 days after BM cell injection.

### 2.9. Competitive BM Transplantation

BMCs were isolated from CD45.2-positive WT and *Pon2*^*-/*-^ mice and mixed 1 : 1 with BMCs isolated from CD45.1 WT mice. Afterwards, 8 × 10^6^ WT CD45.1/WT CD45.2 (control) or WT CD45.1/*Pon2^−/−^* CD45.2 mixed BMCs were intravenously injected into irradiated WT CD45.2 recipient mice. About 50 *μ*l blood from competitive transplanted mice was taken 3, 7, 11, 15, 19, and 22 weeks after injection and stained with CD45.1 and CD45.2 antibodies to detect HSPC engraftment using congenic C57BL/6 mice that differ at the Ly5 locus [41]. Irradiated mice were treated with Borgal for about 4 weeks after irradiation. All gating strategies are shown in Figure S4.

### 2.10. Serial Transplantation of Aged BMCs

5 × 10^6^ BMCs, isolated from aged WT and *Pon2^−/−^* mice, were separately *i.v.* injected into irradiated, young WT recipient mice. 21 days after transplantation, the BMCs were isolated by flushing tibial and femoral bones of the recipient mice and then resuspended in DMEM and once more *i.v.* injected into irradiated, young WT recipient mice. During the next 21 days, the survival rate of recipient mice was determined.

### 2.11. Measurement of Total ROS by H_2_DCF-DA Staining

After BM isolation and staining of HSPCs using cell surface markers as described above, cells were incubated with 0.5 *μ*M fluorescent ROS indicator CM-H_2_DCF-DA (Molecular Probes/Thermo Fisher Scientific, Dreieich, Germany) for 30 min at 37°C. Subsequent to washing of cells using Krebs HEPES buffer (Noxygen, Elzach, Germany), total ROS was assessed by analyzing H_2_DCF-DA signal intensity on a FACSCanto II (BD Biosciences) flow cytometer with BD FACSDiva™ Software (excitation/emission CM-H_2_DCF-DA: 488/520 nm). All gating strategies are shown in Figure S4.

### 2.12. Measurement of ROS Production via L-012

ROS production was determined using the luminol derivative L-012 (8-amino-5-chloro-7-phenylpyridol [3,4-d] pyridazine-1,4 (2H,3H) dione; Wako Chemicals, Richmond, U.S.A.) as previously described for tissue homogenates, whole blood, and isolated leukocytes [42]. Freshly isolated BMCs of WT and *Pon2*^*-/*-^ mice were centrifuged and resuspended in modified Krebs HEPES buffer at a concentration of 1 × 10^7^ cells/ml. 50 *μ*l of cell suspension per well (containing 5 × 10^5^ cells) was loaded in a 96-well plate. Chemiluminescence was recorded after addition of 40 *μ*M L-012 and in some cases 10 *μ*M DMNQ (2,3-dimethoxy-1,4-naphthoquinone), a redox-cycling agent that induces intracellular superoxide anion and hydrogen peroxide formation. L-012 chemiluminescence was measured simultaneously for the two experimental groups for about 75 minutes every 4 minutes using a Microplate Centro LB960 Luminometer (Berthold Technologies, Sprendlingen, Germany). The photon counts were normalized to chemiluminescence of L-012 in modified Krebs HEPES buffer only.

### 2.13. Cell Cycle Analysis of BM Populations

Cell surface staining was performed as described above. Subsequently, samples were incubated in Cytofix/Cytoperm (BD Biosciences) for 15 minutes. Cells were washed using Perm/Wash, resuspended in a buffer containing an anti-Ki-67 antibody (Alexa Fluor 647, diluted 1 : 30 in Perm/Wash), and incubated for at least 30 minutes. After washing with Perm/Wash, cells were resuspended in 100 *μ*l Hoechst 33342 (diluted 1 : 500 in PBS) and incubated for 15 minutes. Analyses were performed using BD LSR II Flow Cytometer, and data were analyzed using BD FACSDiva™ or FlowJo Software. All gating strategies are shown in Figure S5.

### 2.14. Colony-Forming-Unit Assays (CFUs)

3 × 10^4^ BMCs from WT and *Pon2*^−/−^ mice were cultured in MethoCult™ GF M3434 (StemCell Technologies, Cologne, Germany) in accordance with the manufacturer's instructions. 10-12 days after plating, the colonies were quantified and identified using a Leitz DM IL microscope (Leica, Wetzlar, Germany).

### 2.15. Homing

Homing of hematopoietic cells to the bone marrow was analyzed as described in Yusuf and Scadden [43], but the BMCs were isolated by flushing instead of crushing the bones.

### 2.16. Assessing Gamma-H2AX Levels of LSK Cells

Cell surface staining was performed using the lineage cocktail of biotin-conjugated antibodies described above as well as streptavidin (APC-Cy7), Sca-1 (PECy7), and ckit (APC). Cells were fixed and permeabilized using Cytofix/Cytoperm (BD Biosciences) and then stained with *γ*H2AX antibody (Alexa Fluor 488, BioLegend, San Diego, U.S:A.) for 2 hours on ice. Data acquisition was performed on a FACSCanto II (BD Biosciences), and histogram overlay images were created using CellQuest Pro Software (BD Biosciences).

### 2.17. Gene Expression Analyses

According to previous studies, PON2 expression levels are the highest in the lung, intestine, heart, and liver [44]. To prove *Pon2* mRNA expression as well as determine cell-specific *Pon2* expression in HSPCs, we isolated mRNA from FACS-sorted LT-HSCs, ST-HSCs, multipotent progenitor cells (MPPs), common myeloid progenitors (CMPs), granulocyte-macrophage progenitors (GMPs), and megakaryocyte-erythroid progenitors (MEPs) and performed two-step qRT-PCR analyses. To analyze the effect of the redox-cycler DMNQ on CXCR4 mRNA expression, RNA was isolated from HPC7 and BA/F3 cells treated with or without 10 *μ*M DMNQ for 2 to 8 h. The RNA was reverse transcribed using the SuperScript™ VILO™ Master Mix (Invitrogen/Thermo Fisher Scientific, Dreieich, Germany). Then, qPCRs were performed using primers and double-labeled probes (5′‐FAM‐>3′TAMRA; all from Eurofins Genomics, Hamburg Germany; listed in Table S1) or with PrecisionPLUS 2x qPCR MasterMix with SYBR green (Primer Design, Chandler's Ford, United Kingdom) as described by the manufacturer. mRNA expression levels were analyzed according to previously established protocols [45], generally applying 2 housekeeping genes (*Gapdh*, *Actb*; for primer used, see Table S1).

### 2.18. Total RNA Sequencing

For total RNA sequencing (RNA-seq) analyses, Lin^−^, Sca^1+^, ckit^+^, CD135^−^, and CD150^+^ cells (which represent a mixture of LT-HSC and MPP2 cells—for a better discrimination hereafter referred to as HSCs) [46] were isolated from BM of young WT or *Pon2*^−/−^ mice (*n* = 6 each) by FACS as described above. 10 HSCs per well and a total of 8 wells per mouse were FACS sorted into a 96-well plate (8 RNA-seq per mouse and 48 RNA-seq per genotype), containing lysis buffer (Qiagen, Hilden Germany). Subsequently, cells were handed over to the genomics core facility of the Institute of Molecular Biology (Mainz, Germany) for RNA-seq using Smart-seq2-protocol for library preparation and NextSeq® 500/550 High Output Kit v2 (Illumina, Cambridge, United Kingdom) for sequencing. Quality of raw sequencing reads was assessed using FastQC (Babraham Bioinformatics), and adapters were trimmed using Trimmomatic (v0.36 [47]). Raw RNA-seq reads were then mapped to mouse reference genome (gencode release M12 GRCm38.p5) using the STAR aligner (v2.5.3a [48]), with an option of “-quantModeGeneCounts” to count the number of reads mapped per gene. The numbers of high-quality reads were 21.1 to 32.6 million reads. Between 67 and 76% of the reads were aligned to the mouse genome. DESeq2 (v1.18.1 [49]) was used to identify genes differentially expressed after *Pon2* knockout. Genes with fold change higher than 2 and FDR below 0.05 were considered as differentially expressed. Overrepresentation analysis was performed using the ConsensuPathDataBase release 34 [50].

### 2.19. RNA-seq or Microarray Analyses

All analyses of public RNA-seq or microarray data from the literature were performed using the software CLC genomic workbench (version 21.0.03) using parameters as recommended by the manufacturer.

### 2.20. Statistics

GraphPad Prism software (version 9) was used, applying 2-tailed Student's *t*-test (normally distributed data, skewness < 1) for comparison of two groups. For more than 2 groups, 1-way ANOVA with Tukey's multiple comparisons test or 2-way ANOVA with Bonferroni's multiple comparisons test was applied. Numbers of mice in the experimental groups or analyzed numbers of independent cell experiments are indicated in the figures. *P* < 0.05 was considered significant.

## 3. Results

### 3.1. Pon2 mRNA Expression Levels Vary between HSCs and Progenitor Cells in Young and Old Mice

**Figure 1:**
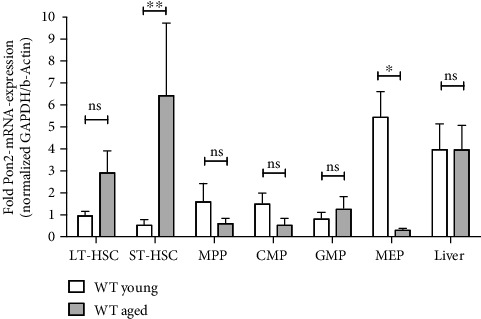
*Pon2* mRNA expression level in WT mice changes upon aging. LT-HSCs, ST-HSCs, MPPs, CMPs, GMPs, MEPs, and liver cells were obtained from young (2-3 months) and aged (>9 months) WT mice. *Pon*2, *Gapdh*, and *Actb* mRNA expression was analyzed by qRT-PCR. *Pon*2 mRNA expression was normalized to *Gapdh* and *Actb* mRNA expression. The relative *Pon*2 mRNA expression in LT-HSCs from young WT mice was set to 1. Shown are the mean + SEM of *n* = 3–6 experiments using 2-6 mice per group (^∗∗^*P* < 0.01, ^∗^*P* < 0.05, ns: not significant vs. WT cells; 2-way ANOVA with Bonferroni's multiple comparisons test).

Pon2 mRNA expression was measured in subsets of BMCs while liver cells, that are known to express high Pon2 mRNA levels, were used as a positive control. Our results (Figure 1) indicate differential, cell-specific Pon2 expression levels between 0.15- and 1.35-fold compared to liver cells as well as changes of Pon2 expression in HSPCs as a function of age. In young animals (10-14 weeks), LT- and ST-HSCs showed low Pon2 mRNA expression levels, which slightly increased in committed progenitor cells (multipotent progenitor cells (MPPs), common myeloid progenitors (CMPs)) and significantly increased in megakaryocyte-erythroid progenitors (MEPs) (Figures 1 and S1A). In contrast, expression analysis in HSPCs of aged animals (>9 months) revealed the lowest Pon2 mRNA levels in MEPs, CMPs, and MPPs, but higher in LT- and ST-HSCs (Figures 1 and S1B). No changes in Pon2 expression as a function of age were observed in hepatic control cells. Analysis of available RNA-seq data sets of HSCs and progenitor cells of young mice (PRJNA603283 [51], PRJNA665066) demonstrated similar patterns of Pon2 mRNA expression as those observed in our experiments (Figures 1 and S1C). Finally, analysis of available RNA-seq (PRJNA524895 [52], PRJNA528500 [53], and PRJNA635499 or microarray data sets (GSE76276) [54]; see Figure S1D) revealed no difference in Pon2 mRNA expression levels between LT-HSCs of young, aged, or old animals.

### 3.2. Young Pon2^−/−^ Mice Show Quantitative Changes in HSPCs but an Unaltered Myeloid/Lymphoid Ratio

**Figure 2:**
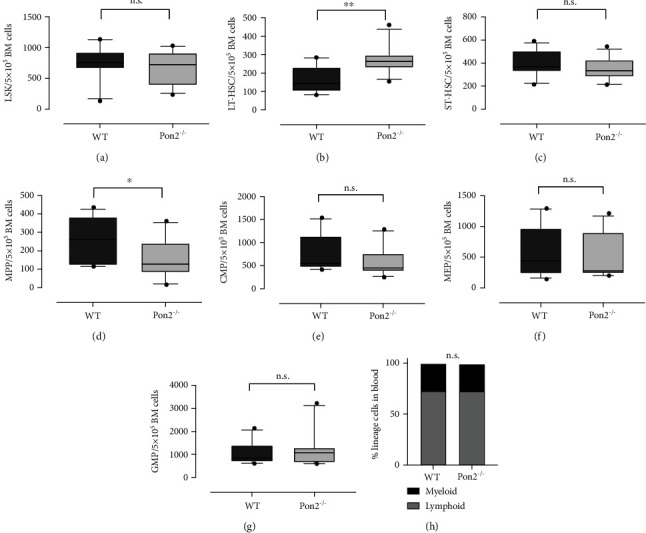
Young *Pon2*^−/−^ mice show quantitative abnormalities in HSPCs but unaltered myeloid/lymphoid ratio. Graphs showing absolute cell numbers per 0.5 10^5^ whole bone marrow cells (WBMC) of (a) LSK cells, (b) LT-HSCs, (c) ST-HSCs, (d) MPPs, (e) CMPs, (f) MEPs, and (g) GMPs of young WT and PON2^−/−^ mice. *n* = 11/group, box and whiskers; whiskers: 10-90 percentile. ^∗∗∗^*P* < 0.001, ^∗∗^*P* < 0.01, ^∗^*P* < 0.05, n.s.: not significant; two-tailed unpaired *t*-test. (h) Percentage of myeloid and lymphoid cells in blood of young WT and PON2^−/−^ mice (*n* = 37–50). n.s.: not significant; two-tailed unpaired *t*-test.

Analysis of different HSPC subpopulations using flow cytometry (Figures 2(a)–2(g)) revealed a significant increase of LT-HSCs and decreased numbers of MPPs in BM of young *Pon2*^−/−^ mice compared to WT animals. No significant changes were observed for total Lin^−^Sca-1^+^c-Kit^+^ (LSK) cells, ST-HSCs, CMPs, GMPs, and MEPs. We also determined the ratio of myeloid and lymphoid cells in the peripheral blood (Figure 2(h)). No difference in the myeloid/lymphoid ratio between WT and *Pon2^−/−^* mice was detected.

### 3.3. Pon2^−/−^ Mice Show Quantitative Changes in Blood Counts

We next tested the consequences of Pon2 deficiency on the peripheral blood populations by performing blood counts of young Pon2^−/−^ and WT mice using a Sysmex Automated Hematology Analyzer.

**Figure 3:**
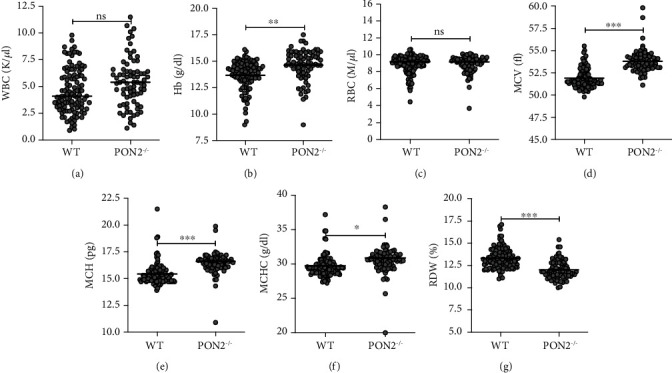
Analysis of peripheral blood from young *Pon2^−/−^* mice compared to WT mice of the same age. Shown are (a) the white blood cell count (WBC), (b) hemoglobin (Hb), (c) red blood cell count (RBC), (d) mean corpuscular volume (MCV), (e) mean corpuscular hemoglobin (MCH), (f) mean corpuscular hemoglobin concentration (MCHC), and (g) red cell distribution width (RDW), in blood of young WT and PON2^−/−^ mice. Dot plot diagram. Median, dot each animal; *n* = 78–113. ^∗^*P* < 0.05, ^∗∗^*P* < 0.01, ^∗∗∗^*P* < 0.001; n.s. = not significant; *t*-test.

Compared with WT, Pon2^−/−^ mice had similar leukocyte (WBC, Figure 3(a)) and erythrocyte (RBC, Figure 3(c)) counts, but increased hemoglobin (Hb, Figure 3(b)). Furthermore, Pon2^−/−^ erythrocytes displayed several qualitative abnormalities, such as enhanced mean corpuscular volume (MCV, Figure 3(d)), mean corpuscular hemoglobin (MCH, Figure 3(e)), and mean corpuscular hemoglobin concentration (MCHC, Figure 3(f)) and reduced red cell width distribution (RWD, Figure 3(g)).

### 3.4. Pon2 Deficiency Associates with a Bias towards Erythropoiesis Both in Physiological and Stress Conditions

**Figure 4:**
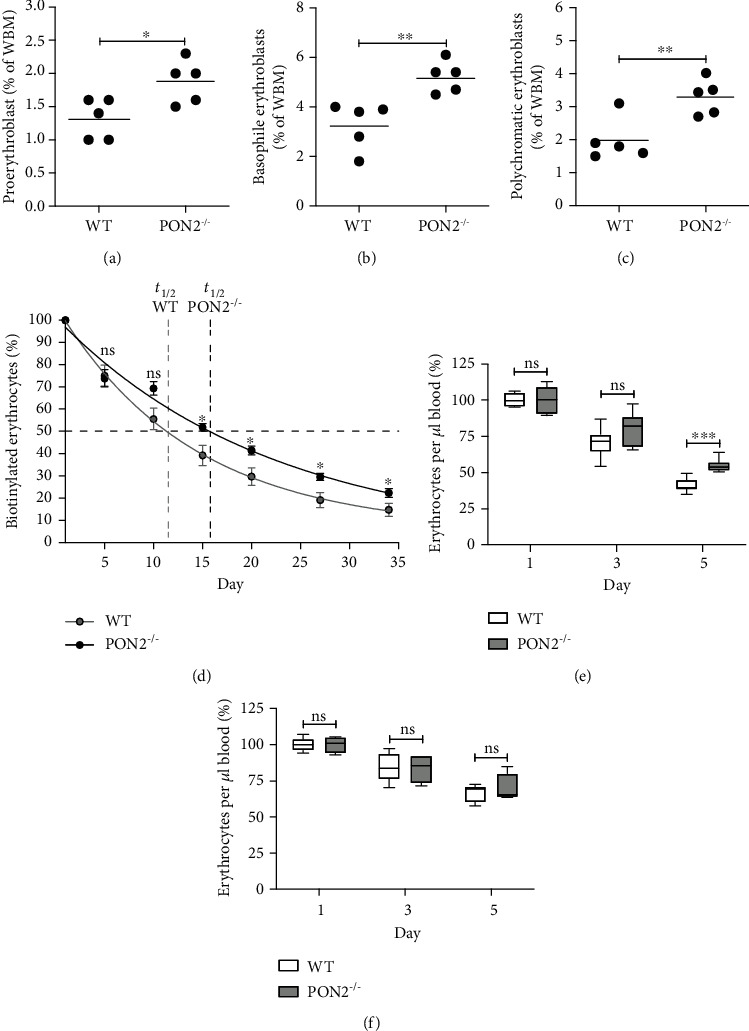
Effects of PON2-inactivation on erythropoiesis. (a–c) Analysis of 3 different stages of erythrocyte development in the bone marrow of WT and Pon2^−/−^ mice. Following the isolation of the bone marrow, cells were stained with the antibodies CD71 and Ter119 and analyzed by flow cytometry. The graphs show the percentage of (a) proerythroblasts (CD71 high/Ter119 mid), (b) basophilic erythroblasts (CD71 high/Ter119 high), and (c) polychromatic erythroblasts (CD71 mid/Ter119 mid). Dot plot diagram. Dot each individual animal; *n* = 5. ^∗^*P* < 0.05, ^∗∗^*P* < 0.01; *t*-test. (d) Lifespan of erythrocytes in young PON2^−/−^ mice. Erythrocyte degradation and regeneration were analyzed using in vivo biotinylation. Young *Pon2*-deficient and WT mice were injected with sulfo-NHS-biotin (intravenous), and small blood samples were taken every few days over a total of 34 days. Afterwards, isolated blood cells were stained with fluorescence-conjugated streptavidin as well as the erythrocyte specific marker Ter119 and analyzed using flow cytometry to determine the number of biotinylated erythrocytes. Results are shown as percentage of biotinylated erythrocytes at day 1, immediately after biotin injection; mean ± SEM, *n* = 7–8. A nonlinear regression was calculated (R2 erythrocytes = 0.90); vertical dashed lines represent the half-life (*t*_1/2_) of the respective cells from WT (light gray) or *Pon2^−/−^* (black) mice. ^∗^*P* < 0.05; n.s.: not significant; *t*-test. (e, f) Percentage of red blood cells in the blood of Pon2^−/−^ and WT mice during the induction of hemolytic anemia by phenylhydrazine. *Pon2*-deficient and wild-type mice were i.p. injected with either phenylhydrazine or PBS (control group) on days 1 and 3. On day 1 (before the start of treatment) and on days 3 and 5, a small amount of blood was taken from the test animals by scratching the *Vena caudalis mediana* in order to analyze the number of erythrocytes. The percentage of erythrocytes in the blood (e) of the phenylhydrazine group and (f) of the control group is shown in comparison to the mean value of the respective starting amount. *n* = 4–8; mean ± SEM. ^∗∗∗^*P* < 0.001; ns = not significant; *t*-test.

In opposition with bone marrow HSPC subpopulations, in which abnormalities were moderate and limited to LT-HSCs and MPPs only, peripheral blood of *Pon2*-deficient mice had severe erythroid irregularities. We therefore next tested whether the Pon2^−/−^-associated erythroid progenitors are likewise subject to numerical aberrancies. Indeed, as shown in Figures 4(a)–4(c), flow cytometry staining analyses of erythroblasts of *Pon2^−/−^* BMs demonstrated increased counts at all stages of differentiation. We also analyzed the lifespan of erythroid cells using an *in vivo* biotinylation assay coupled to flow cytometry. Of importance, Ter119-positive erythroid cells from *Pon2^−/−^* mice had a significantly enhanced lifespan (*t*_1/2_ = 16 days) compared to those in WT controls (*t*_1/2_ = 11 days) (Figure 4(d)).

Finally, we assessed the dynamics of stress erythropoiesis in *Pon2^−/−^* and WT mice after induction of hemolysis via intraperitoneal application of phenylhydrazine or the control substance PBS. Phenylhydrazine caused a strong decrease of erythrocyte counts in WT mice by day 5 posttreatment commencement (Figure 4(e)). In contrast, the expected hemolysis was weaker in Pon2^−/−^ mice, in which the percentage of erythrocytes in blood remained significantly higher (Figure 4(e)). In control-treated animals, no significant changes between WT and *Pon2^−/−^* animals were seen (Figure 4(f)).

Altogether, these data demonstrate a specific role for Pon2 to limit erythroid commitment and lifespan.

### 3.5. In Young Pon2^−/−^ Mice, Reciprocal BM Transplantation Reveals Cell Intrinsic as well as Extrinsic Phenotypes

We next aimed to assess the functional importance of the observed quantitative changes in HSPC compartments. To determine whether the effects of *Pon2* deficiency on HSPCs are cell intrinsic or niche derived, we performed reciprocal BM transplantations (see Figure S2A), resulting in four experimental groups: WT recipient (r)/WT donor (d) and *Pon2*^−/−^(r)/*Pon2*^−/−^(d) as well as chimeras comprising *Pon2*^−/−^(r)/WT(d) and WT(r)/*Pon2*^−/−^(d). Successful reconstitution or depletion of Pon2 in hematopoietic cells of recipient mice was verified by qRT-PCR (Figure S2B). Subsequent flow cytometric analyses of HSPC count showed cell-specific and mixed effects (cell specific + niche effect). Reciprocal BM transplantation revealed increased LT-HSC, CMP, and MEP numbers (Figures S2C, S2F, and S2G) in the *Pon2*^−/−^ animals receiving *Pon2*^−/−^ BM. No major differences were seen in the chimeras, which indicate complementary cell intrinsic and extrinsic effects.

### 3.6. Pon2 Deficiency in Young Mice Leads to Oxidative Stress, but Does Not Induce Apoptosis or Cause DNA Double-Strand Breaks

**Figure 5:**
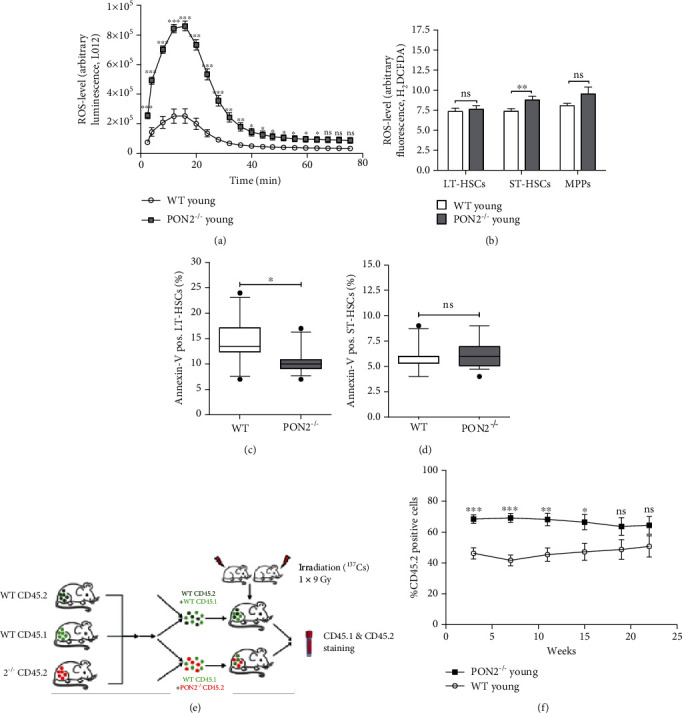
PON2 deficiency causes oxidative stress, but does not impair but rather improve HSC function. (a) L-012 chemiluminescence signal of freshly isolated BMCs from young WT and *Pon2*^−/−^ mice quantified over time for ROS formation (representative graph); mean ± SEM. ^∗^*P* < 0.05, ^∗∗^*P* < 0.01, ^∗∗∗^*P* < 0.001, ns: not significant vs. WT; two-tailed unpaired *t*-test. (b) Total ROS level in LT-HSCs, ST-HSCs, and MPPs of young (2-3 months) WT and *Pon2*^−/−^ mice stained with cell-specific markers (LT-HSCs: Lin^−^, Sca^1+^, ckit^+^, CD135^−^, and CD150^+^; ST-HSCs: Lin^−^, Sca^1+^, ckit^+^, CD135^−^, and CD150^−^; MPPs: Lin^−^, Sca^1+^, ckit^+^, CD135^+^, and CD150^−^) and H_2_DCF-DA, analyzed by FACS (*n* = 12–13); mean + SEM. ^∗∗^*P* < 0.01, ns: not significant vs. WT; two-tailed unpaired *t*-test (representative histograms showing the DCF-DA data comparing WT and Pon2^−/−^ mice, see Figure S6). BMCs isolated from young WT and *Pon2*^−/−^ mice stained with cell-specific markers for (c) LT-HSCs or (d) ST-HSCs and annexin V for quantification of apoptotic cells (*n* = 12–16). Box and whiskers; whiskers: 10-90 percentile; ^∗^*P* < 0.05, ns: not significant vs. WT; two-tailed unpaired *t*-test. (e) Experimental scheme for competitive bone marrow transplantation. (f) Percentage of CD45.2-positive cells in the blood of competitive transplanted young mice 3, 7, 11, 15, 19, and 22 weeks after transplantation (*n* = 9); mean ± SEM. ^∗^*P* ≤ 0.05, ^∗∗^*P* ≤ 0.01, ^∗∗∗^*P* < 0.001, ns: not significant; two-tailed unpaired *t*-test.

Using two approaches to analyze basal ROS production, we examined murine BMCs under steady-state conditions in *Pon2*^−/−^ mice compared to WT animals. First, we analyzed superoxide/hydrogen peroxide in BMC using detection with the chemiluminescent probe L-012. In accordance with previously published inhibition of superoxide/hydrogen peroxide production by PON2 [10], we detected markedly increased L-012 chemiluminescence signals in BMCs of young *Pon2*^−/−^ mice compared to WT mice (Figure 5(a)). Furthermore, we analyzed total ROS levels in WT or *Pon2*^−/−^ HSPCs with the fluorescent dye CM-H_2_DCF-DA by flow cytometry. The measurements demonstrated significantly enhanced ROS formation in *Pon2*^−/−^ ST-HSCs and numerically enhanced ROS in *Pon2*^−/−^ MPPs of young animals (Figure 5(b)). Interestingly, no differences were observed in LT-HSCs.

We hypothesized that PON2 deficiency leads to increased apoptotic cell death in HSCs. Using annexin V staining, we analyzed the percentage of apoptotic LT- and ST-HSCs in BM of *Pon2*^−/−^ and WT mice by flow cytometry. These measurements revealed significantly less annexin V-positive LT-HSCs but no differences in annexin V-positive ST-HSCs in young *Pon2*^−/−^ mice compared to WT (Figures 5(c) and 5(d)). We also detected no changes in fluorescence signal intensity of gamma-H2AX antibody in *Pon2*-deficient LSK cells compared to WT cells (Figure S3A), indicating genetic stability in WT and *Pon2*^−/−^ mice, despite higher ROS levels in *Pon2*^−/−^ mice.

### 3.7. Young Pon2^−/−^ BM Cells Outcompete WT Cells at Early but Not at Later Time Points

To analyze whether the increased number of LT-HSCs in *Pon2*^−/−^animals confers increased fitness, we performed competitive BM transplantation assays. Engraftment of WT and *Pon2*^−/−^BMCs was analyzed by flow cytometry using the cell surface markers CD45.1 and CD45.2, respectively (Figure 5(e)) as described [41]. Competitive transplantation of WT and *Pon2*^−/−^BMCs in a 1 : 1 ratio revealed significant advantages of *Pon2*^−/−^ BMCs in multilineage reconstitution at early time points (Figure 5(f)) whereas engraftment was similar between WT and *Pon2^−/−^* cells after week 15.

### 3.8. Young Pon2^−/−^ and WT HSPCs Show No Differences in Cell Cycle Status, Colony-Forming Ability, and Homing

Since increased ROS level disrupts the quiescent state of HSCs and can stimulate them to proliferate and differentiate [18], we performed cell cycle analysis using Hoechst 33342 and Ki-67 to distinguish between cells in G0- and G1- as well as G2-, S-, and M-phase. We detected no difference in cell cycle status of *Pon2*^−/−^ LSK cells or LT-HSCs compared to WT cells (Figures S4A and S4B). Similarly, colony-forming assays revealed no difference in colony-forming ability of WT or *Pon2*^−/−^ BMCs after 10 days of incubation. Besides the equal amounts of total colonies (Figure S4C), there was also no change in the number of specific colonies (Figure S4D), indicating no Pon2-mediated impact on colony forming and differentiation.

We also analyzed the homing ability of BMCs. We examined homing efficiency using the fluorescent dye DiI and analyzing BM of lethally irradiated recipient mice 48 h after injecting stained *Pon2*^−/−^ or WT BMCs (Figure S4E). Flow cytometric analysis revealed no differences in homing ability of WT and *Pon2*^−/−^ BMCs (Figure S3F). Therefore, we speculate that the higher frequency of *Pon2^−/−^*-derived cells upon competitive BM transplantation at early time points (Figure 5(f)) was likely caused by the increased number of LT-HSCs in *Pon2^−/−^*-derived BM cells (Figure 2(b)) and not due to increased fitness of *Pon2^−/−^* HSCs.

### 3.9. Aged Pon2^−/−^ Mice Reiterate the Increased LT-HSCs Proportion, but Also Exhibit Changes Leading to an Altered Myeloid/Lymphoid Ratio

**Figure 6:**
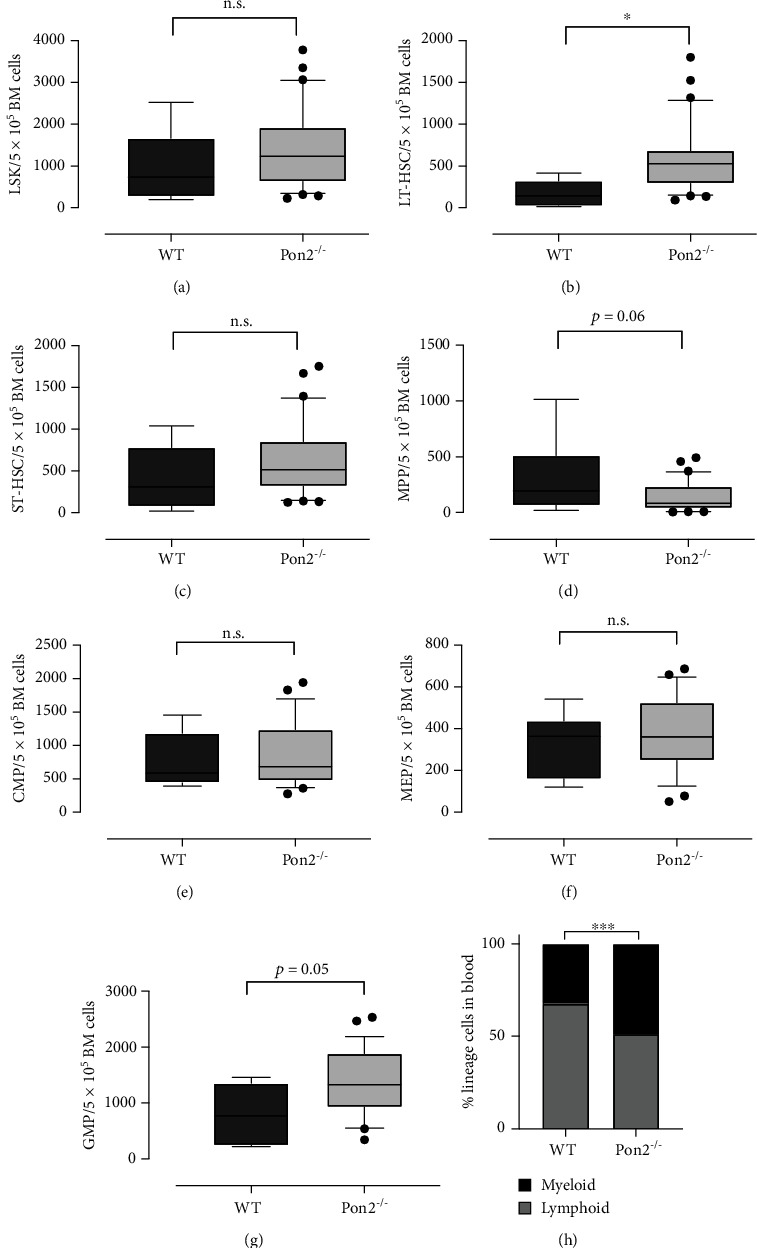
Aged *Pon2*^−/−^ mice show increased cell count of HSPCs and altered myeloid/lymphoid ratio in the blood. Graphs showing absolute cell numbers per 0.5 10^5^ whole bone morrow cells (WBMC) of (a) LSK cells, (b) LT-HSCs, (c) ST-HSCs, (d) MPPs, (e) CMPs, (f) MEPs, and (g) GMPs of old WT and PON2^−/−^ mice. *n* = 6–30, box and whiskers; whiskers: 10-90 percentile. ^∗∗∗^*P* < 0.001, ^∗∗^*P* < 0.01, ^∗^*P* < 0.05, n.s.: not significant; two-tailed unpaired *t*-test. (h) Percentage of myeloid and lymphoid cells in the blood of aged WT and Pon2^−/−^ mice (*n* = 37–50). ^∗∗∗^*P* < 0.001 vs. WT; two-tailed unpaired *t*-test.

In accordance with the analysis in young mice, flow cytometry on BMC of aged *Pon2*^−/−^ mice demonstrated an increase of LT-HSCs. Additionally, a surprising increase in the proportion of GMPs was noted, while all other committed progenitors remained at comparable levels with those in control mice (Figures 6(a)–6(g)). These increased GMP levels indicate potential intensified myeloid skewing compared to age-matched WT mice, which could be confirmed by a significantly shifted myeloid/lymphoid ratio in the peripheral blood of aged *Pon2*^−/−^ mice (Figure 6(h)).

### 3.10. Similar Total ROS Levels in Old Pon2^−/−^ HSCs Compared to WT HSCs, but Significantly Decreased Baseline Apoptosis

**Figure 7:**
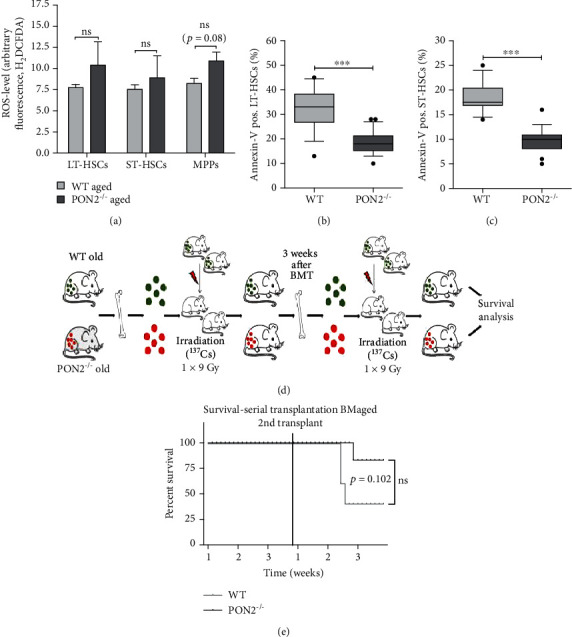
Aged *Pon2*^−/−^ mice show numeric increased total ROS level in HSPCs, but significantly decreased apoptotic rate and increased functionality. (a) Total ROS level in LT-HSCs, ST-HSCs, and MPPs of aged (≥9 months) WT and *Pon2*^−/−^ mice stained with cell-specific markers (LT-HSCs: Lin^−^, Sca^1+^, ckit^+^, CD135^−^, and CD150^+^; ST-HSCs: Lin^−^, Sca^1+^, ckit^+^, CD135^−^, and CD150^−^; MPPs: Lin^−^, Sca^1+^, ckit^+^, CD135^+^, and CD150^−^) and H_2_DCF-DA, analyzed by FACS (*n* = 3); mean + SEM, ns: not significant vs. WT; two-tailed unpaired *t*-test. BMCs isolated from aged WT and *Pon2*^−/−^ mice stained with cell surface markers for (b) LT-HSCs or (c) ST-HSCs and annexin V for quantification of apoptotic cells (*n* = 14–29); box and whiskers; whiskers: 10-90 percentile. ^∗∗∗^*P* < 0.001; two-tailed unpaired *t*-test. (d) Experimental scheme for serial transplantation of aged BMCs. (e) Percent survival of mice after serial transplantation of aged WT or *Pon2*^−/−^ BMCs (*n* = 5–6); ns: not significant vs. WT; survival rates are shown as a Kaplan-Meier plot; log rank (Mantel-Cox) test (*P* = 0.102).

Total ROS in HSPCs of aged *Pon2*^−/−^ mice was assessed by flow cytometry using H_2_DCF-DA staining. Similar to young *Pon2^−/−^* HSCs, no major differences in ROS levels were observed in all subpopulations of the LSK fraction compared to WT cells (Figure 7(a)). Again, the number of annexin V-positive cells was significantly decreased in *Pon2^−/−^*LT- and ST-HSCs (Figures 5(b) and 5(c)), whereas DNA damage analyses did not reveal higher levels of DNA double-strand breaks compared to WT cells (Figure S3B).

### 3.11. In Serial Transplantation Experiments, Recipients of BM from Aged Pon2^−/−^ Mice Show No Reduced Survival Rate

To analyze the functionality of aged *Pon2*^−/−^ BMC, we performed serial transplantation experiments to induce decent proliferative stress. The BMCs were isolated from aged WT and *Pon2*^−/−^ mice. HSCs from aged animals have been described to have a reduced ability to repopulate recipient mice in *in vivo* transplantation assays [55]. Therefore, we performed 2 rounds of transplantations (Figure 7(d)). Of note, aiming to create a condition of higher stress levels upon the bone marrow repopulation, we shortened the time between the primary and secondary retransplantation to 3 weeks. These experiments revealed no statistical differences in the survival rate of the animals transplanted with *Pon2*^−/−^ or WT BMCs, albeit at a low sample size (5 vs. 6 animals). However, and in line with the observed increased number of LT-HSCs and decreased levels of apoptosis, more recipient mice transplanted with BMCs of aged *Pon2*^−/−^ donors were alive at day 21 of the second transplantation round compared to recipients of BMCs from wild-type animals (Figure 7(e)).

### 3.12. RNA-seq Analyses Show Enhancement of the Expression of Survival Genes in HSCs of Young Pon2^−/−^ Mice

**Figure 8:**
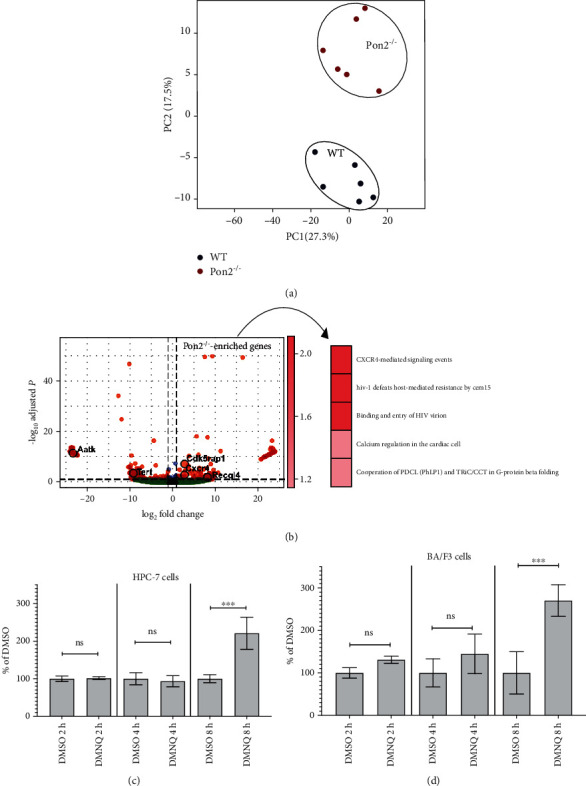
Cxcr4 and Cxcr4 pathway-related genes are upregulated in young Pon2^−/−^ HSCs and upon treatment with DMNQ in murine hematopoietic stem cell lines. (a) PCA plot displaying the variance of gene expression in 5 *Pon2*^−/−^ HSCs and 5 matched WT LT-HSCs. (b) Left panel: volcano plot of gene expression differences between *Pon2*^−/−^ and matched WT HSCs. Genes whose expression is significantly upregulated in *Pon2*^−/−^ LT-HSCs are marked in red, while genes whose expression is significantly downregulated in *Pon2*^−/−^ HSCs are marked in blue. Right panel: heat map showing the top 5 overrepresented pathways in *Pon2*^−/−^ HSCs. The scale was calculated as -log_10_ FDRq. (c) HPC-7 or (d) BA/F3 cells were treated with *DMSO* (control) or 10 *μ*M *DMNQ* for 2 to 8 h. *CRCR4* and *Gapdh* mRNA expression was analyzed by qRT-PCR. *CXCR*4 mRNA expression was normalized to *Gapdh* mRNA expression. The relative *CXCR4* mRNA levels in DMSO-treated cells were set to 100%. Shown are the mean ± SEM of *n* = 3–6 experiments (^∗∗∗^*P* < 0.001, ns: not significant vs. DMSO-treated cells; two-sided *t*-test).

Although increased ROS levels in progenitor cells, in particular in aged mice, caused a premature aging phenotype with increased frequencies of myeloid progenitors and a shifted myeloid-to-lymphoid ratio in the peripheral blood of aged *Pon2*^−/−^ mice, we did not observe an exhaustion phenotype under conditions of stress. This observation might be the consequence of increased LT-HSC numbers or the reduction of baseline apoptotic cell death in Pon2^−/−^ LT-HSCs. We hypothesized that depletion of Pon2 induces a compensatory program during the earliest hematopoietic stages to overcome the harmful effects of supraphysiological ROS levels that we discovered in whole BM. To address this hypothesis, we performed RNA-seq analyses of Lin^−^, Sca^1+^, ckit^+^, CD135^−^, and CD150^+^ cells (representative for a mixture of LT-HSCs and MPP2—hereafter referred to as HSC) isolated from young WT or *Pon2*^−/−^ mice (*n* = 6 per genotype). The comparison of whole transcriptomes of HSCs isolated from young WT or *Pon2*^−/−^ mice using DESeq2 identified 341 differentially expressed genes. Of these, 168 genes were downregulated and 157 genes were upregulated in *Pon2*^−/−^ HSCs compared to WT HSCs (see Table S2 and Figure 6(b)). Sample distance analysis (see Figure 8(a)) revealed no distinct clustering between the *Pon2*^−/−^ and WT groups, and this coincided with similar spreading profiles in the first principal component (PC1) of the principal component analysis (PCA). Nonetheless, *Pon2*^−/−^ and WT groups clearly clustered separately in the second principal component (PC2), which led us to believe that a rather discrete number of cellular processes/pathways differ between HSCs of young *Pon2*^−/−^ and WT animals.

We noted differential expression of several important regulators of cell survival, such as Telomerase (*Tert*) [56, 57] or the NRF2 pathway genes *Nfe2l2* and *Abcc2* [58, 59] and CXCR4 involved in the regulation of homing, quiescence/proliferation, or migration [60]. Of note, overrepresentation analysis of genes that were upregulated in Pon2^−/−^ HSCs demonstrated significant enrichment of only a discrete number of pathways (Figure 8(b)). Most importantly, the CXCR4-mediated signaling event pathway reached the highest level of significance (FDRq 0.02) among the whole database (Figure 6(b); Tables S3 and S4). Moreover, treatment with the ROS generator DMNQ resulted in enhanced (around 2.5-fold) CXCR4 mRNA expression in HPC-7 hematopoietic stem cells and BA/F3 pro B cells (see Figures 8(c) and 8(d)).

## 4. Discussion

In this study, we comprehensively investigated the function of PON2 in the hematopoietic system. PON2 has been linked to therapy resistance and poor prognosis in different types of leukemia, but the biological function of PON2 in hematopoiesis has not been investigated. Our *in vivo* studies demonstrate that PON2 is involved in the regulation of normal hematopoiesis. First, we determined Pon2 mRNA expression levels in different HSPC subpopulations of young (<3 months) and aged (>9 months) mice. In young mice, Pon2 mRNA levels were increased in committed progenitor cells, in particular in CMPs and MEPs, compared to the HSC compartment. Surprisingly, but at least in line with the high expression levels putatively linked to a specific functional importance in MEPs, Pon2 deficiency in young mice associated with a propensity to a more robust erythropoiesis both in physiological and stress conditions. While this remains to be demonstrated in subsequent works, we believe that Pon2 deficiency might activate an (over) compensatory mechanism to maintain the erythropoiesis.

Meanwhile, expression levels of Pon2 declined with proceeding differentiation in aged mice. Interestingly, in aged mice, depletion of *Pon2* caused an increase of GMPs accompanied by a skewed myeloid-to-lymphoid ratio pointing to at least partly accelerated aging of *Pon2*^−/−^ HSCs in comparison to WT cells. An age-related expansion of different HSPC subpopulations has been described in different HSC aging studies [55, 61]. The enhanced myeloid skewing in aged *Pon2*^−/−^ mice may be caused by a small increase in ROS levels as detected in MPPs [62, 63].

Since studies on cell culture models after PON2 knockdown as well as *Pon2*^−/−^ endothelial cells have shown enhanced ROS formation [12, 13], we determined total ROS level and superoxide/hydrogen peroxide level in BMCs of young animals. Due to their low metabolic activity, HSCs are vulnerable to cellular damage caused by oxidative stress. In physiological quantities, ROS act as signal molecules that regulate stem cell proliferation, differentiation, and mobilization. Even a comparatively minor increase of ROS in HSCs can lead to the malfunction of self-renewal activity and HSC senescence, which can cause premature exhaustion of the HSC pool and hematopoietic dysfunctions [64]. Analysis of superoxide/hydrogen peroxide production showed markedly enhanced formation rates in whole BMC of young *Pon2*^−/−^ animals. However, total ROS levels were not affected in LT-HSCs in young and old Pon2^−/−^ animals. PON2 has been shown to reduce superoxide production mainly at the inner membrane of mitochondria [10], but LT-HSCs demand low amounts of energy, which are almost entirely produced via glycolysis [19]. The increase of mitochondrial superoxide production in *Pon2^−/−^* LT-HSC may therefore be minimal or not even existent. Meanwhile, already with the first step of differentiation into cycling cells—that of ST-HSCs—cells shift from glycolysis to mitochondrial ATP production [20]. The increase of superoxide/hydrogen peroxide (e.g., in ST-HSCs and numerically in MPPs) likely reflects higher ROS level in *Pon2^−/−^* progenitor cells, finally contributing to the observed phenotype resembling premature aging. A mechanism involved in HSC aging is reduction of the activity of the telomerase enzyme, which leads to a limitation of cell proliferation in HSCs [26]. In our studies, we observed a reduction of *Tert* gene expression in HSC isolated from young *Pon2*^−/−^ mice.

Interestingly, quantitative analysis of different HSPC subpopulations in BM of WT and *Pon2^−/−^* mice revealed an increased percentage of LT-HSCs. Further, we observed an advantage over WT-BMCs in competitive repopulation assays at early time points in young animals, likely due to the increased percentage of *Pon2^−/−^* LT-HSCs as a consequence of diminished apoptotic cell death in young and old *Pon2^−/−^* LT-HSCs. Interestingly, gene inactivation of the enzymatically active subunit of the NADPH oxidase holoenzyme subunit gp91phox (NOX2) resulting in reduced ROS generation produced an opposite result. In competitive transplantation with WBM cells from NOX2^−/−^ animals, a reduced engraftment was seen [17]. These data indicate that Pon2-dependent mechanisms additional to ROS detoxification might be involved in mediating the observed phenotype. RNA-seq analyses in HSCs of young animals identified a number of differentially expressed genes described to regulate cell death and proliferation. For example, we observed enhanced expression of the ATP-dependent DNA helicase Q4 (Recql4, DNA repair), the makorin ring finger protein 2 (Mkrn2, antiapoptotic), and the cyclin-dependent kinase 5 regulatory subunit-associated protein 1 (Cdk5rap1) as well as decreased levels of apoptosis-associated tyrosine kinase (Aatk, proapoptotic). RECQL4 is essentially involved in DNA-repair [65, 66] and inactivation of this gene resulted in bone marrow failure due to increased apoptotic rates [67]. In primary leukemia cells and in different leukemia cell lines, enhanced MKRN2 expression results in reduced apoptotic rates and enhanced cell proliferation [68]. In human breast cancer MCF-7 cells, CDK5RAP1 deficiency induces cell cycle arrest and apoptosis indicating an antiapoptotic function of this protein [69]. AATK has been described to be important for the induction of growth arrest and/or apoptosis of myeloid precursor cells [70]. In previous studies, PON2 has been shown to induce antiapoptotic properties [71, 72]. We speculate that in our *Pon2^−/−^* knockout model, a positive feedback is activated at the LT-HSC levels to compensate the increased demand of progenitor cells and accelerated aging phenotype resulting in an increased number of LT-HSCs. In addition to the above-mentioned genes, we detected a significant induction of the expression of *Cxcr*4. The CXCL12/CXCR4 axis is involved in the regulation of homing, quiescence/proliferation, or migration [60]. In young mice with inactivated aryl hydrocarbon receptor (*Ahr*), a small but significant enhancement of the ROS production was seen, similar upon *Pon2* depletion. In microarray experiments, the authors detected a 2.91-fold enhancement of the Cxcr4 expression (*P* value 0.045) [54]. Moreover, treatment with the ROS generator DMNQ resulted in enhanced CXCR4 mRNA expression in HPC-7 hematopoietic stem cells and BA/F3 pro B cells. One important mechanism of CXCL12/CXCR4 signaling in the maintenance of HSC homeostasis is the protection against (oxidative) stress [73]. So it is likely that the enhanced *Cxcr*4 expression in *Pon2*^−/−^ HSC protects these cells from transplantation-induced stress at early time points. In line with previous data [73], this finding allows us to speculate that the CXCR4/CXCL12 axis is upregulated as a result of increased ROS to counteract hematopoietic stem cell exhaustion upon *Pon2* loss. While Pon2 depletion causes increased ROS and cell exhaustion in more mature progenitor cells of young mice, compensatory upregulation of Cxcr4 may protect LT-HSCs of these mice, leading to increased cell numbers and decreased apoptosis providing adequate supply of committed progenitors, a hypothesis that remains to be explored in future investigations.

## 5. Conclusion

In conclusion, our current data indicate that PON2 is involved in the regulation of HSC functions. Enhanced ROS levels in *Pon2^−/−^* progenitor cells correlate with increased frequencies of CMPs and GMPs as well as a skewed myeloid-to-lymphoid ratio in aged mice. Loss of *Pon2* activated an antiapoptotic program in LT-HSCs but also caused increased expression of genes involved in stem cell maintenance, e.g., Cxcr4, Recql4, and Aatk. We speculate that the induction of a “maintenance” program upon *Pon2* depletion counteracts a ROS-mediated premature aging phenotype and ensures proper supply of committed progenitor cells in aged mice. However, further experiments are required to address this hypothesis.

## Data Availability

Raw RNA-seq data are available via the gene expression omnibus (GEO) repository [74] by accession number GSE122553.

## Supplementary Materials

Supplementary Materials Figure S1: *Pon2* mRNA expression in different bone marrow cells of WT mice. Figure S2: in young *Pon2*^−/−^ mice, reciprocal BM transplantation reveals cell intrinsic as well as extrinsic phenotypes. Figure S3: *Pon2*^−/−^ BMCs show no enhanced amount of DNA double-stand breaks in LSK cells. Figure S4: bone marrow cells of young *Pon2*^−/−^ and WT mice show no differences in cell cycle status, colony-forming ability, and homing. Figure S5: gating strategies. Figure S6: representative histograms showing the DCF-DA data of LT- and ST-HSC as well as MPP isolated from young WT and Pon2^−/−^ mice. Table S1: sequences of primers and probes used for qRT-PCR-based quantification of *Pon2* mRNA expression. Table S2: differentially expressed genes identified using DESeq2 and whole genome RNA-seq data from HSCs isolated from WT and *Pon2^−/−^* animals. Table S3: pathways enriched in HSCs isolated from *Pon2^−/−^* animals. Table S4: pathways decreased in HSCs isolated from *Pon2^−/−^* animals.
