# Supplementary material for: Deficiency of Antioxidative Paraoxonase 2 (Pon2) Leads to Increased Number of Phenotypic LT-HSCs and Disturbed Erythropoiesis
Source: Oxid Med Cell Longev. 2021 Jun 25;2021:3917028. doi: 10.1155/2021/3917028 (PMC8253644; doi:10.1155/2021/3917028)
Supplement: Supplementary Materials — Figure S1: Pon2 mRNA expression in different bone marrow cells of WT mice. Figure S2: in young Pon2−/− mice, reciprocal BM transplantation reveals cell intrinsic as well as extrinsic phenotypes. Figure S3: Pon2−/− BMCs show no enhanced amount of DNA double-stand breaks in LSK cells. Figure S4: bone marrow cells of young Pon2−/− and WT mice show no differences in cell cycle status, colony-forming ability, and homing. Figure S5: gating strategies. Figure S6: representative histograms showing the DCF-DA data of LT- and ST-HSC as well as MPP isolated from young WT and Pon2−/− mice. Table S1: sequences of primers and probes used for qRT-PCR-based quantification of Pon2 mRNA expression. Table S2: differentially expressed genes identified using DESeq2 and whole genome RNA-seq data from HSCs isolated from WT and Pon2−/− animals. Table S3: pathways enriched in HSCs isolated from Pon2−/− animals. Table S4: pathways decreased in HSCs isolated from Pon2−/− animals. [file 3917028.f1.pdf]

SUPPLEMENTAL INFORMATIONS

Supplemental Figures with legends

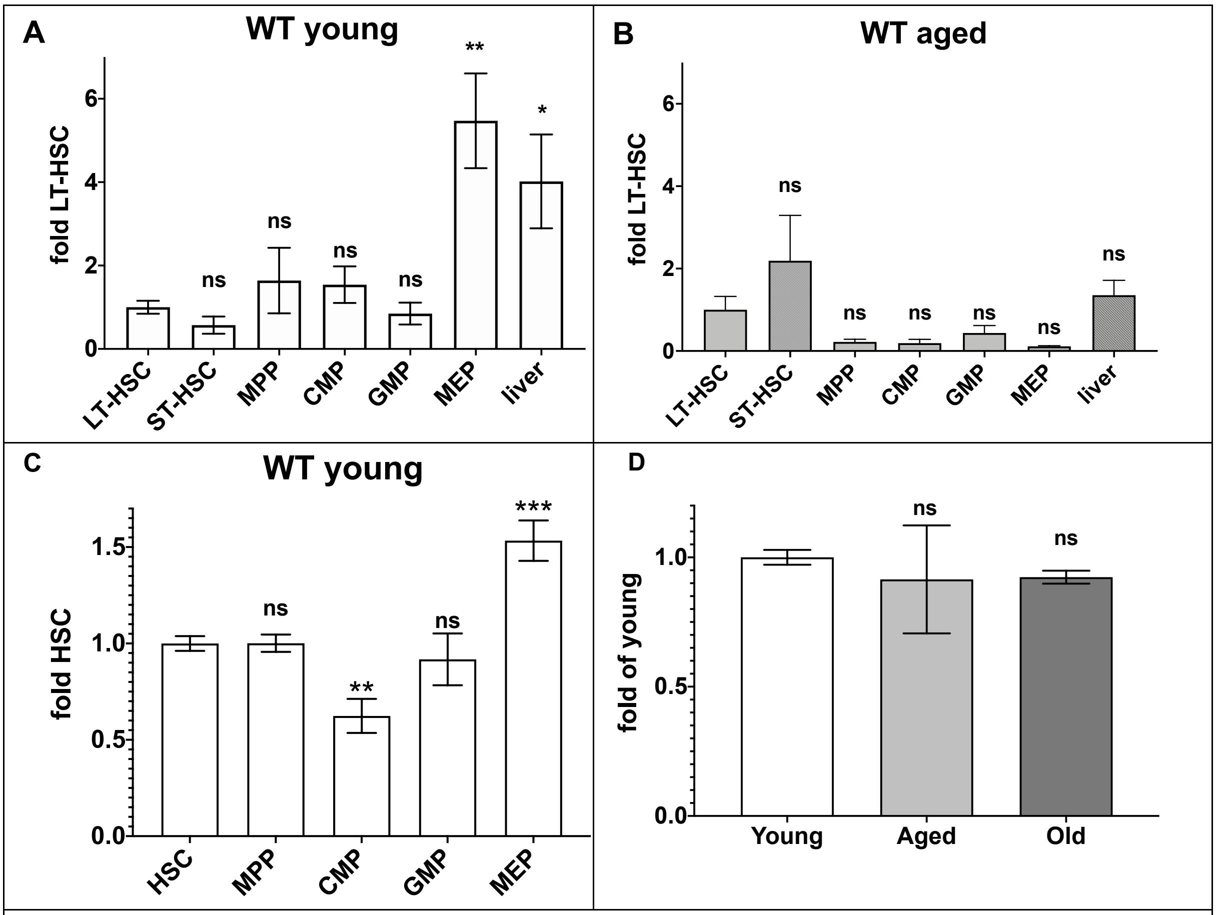

Figure S1. *Pon2* mRNA expression in different bone marrow cells of WT mice

A and B: LT-, ST-HSCs, MPPs, CMPs, GMPs, MEPs and liver cells were obtained from young (2-3 months) and aged (>9 months) WT mice. *Pon*2-, *Gapdh*- and *Actb* mRNA expression was analyzed by qRT-PCR. *Pon*2 mRNA expression was normalized to *Gapdh* and *Actb*-mRNA expression. The relative *Pon*2 mRNA expression in LT-HSCs from young or aged WT mice was set to 1. Shown are the mean ± SEM of n=3-6 experiments using 2-6 mice per group (**p < 0.01, *p < 0.05, ns not significant vs. LS-HSC cells; 1-way Anova with Dunnett's multiple comparisons test).

C: RNA-Seq reads from bioproject PRJNA631793 and PRJNA665066 were analyzed for *Pon*2 gene expression. The values (rpkm) of the HSC of young mice were set as 1fold. Shown are the mean ± SEM (*** p < 0.001 ** p < 0.01, ns not significant vs HSC; 1-way Anova with Dunnett's multiple comparisons test).

D: RNA-Seq reads from bioproject PRJNA524895, PRJNA528500 and PRJNA635499 and Microarray-Data from GSE76276; were analyzed for *Pon*2 gene expression. The values (rpkm or normalized intensities) of the young mice were set as 100 %. Shown are the

mean ± SEM (ns not significant vs. LT-HSC cells form young mice; 1-way Anova with Dunnett's multiple comparisons test).

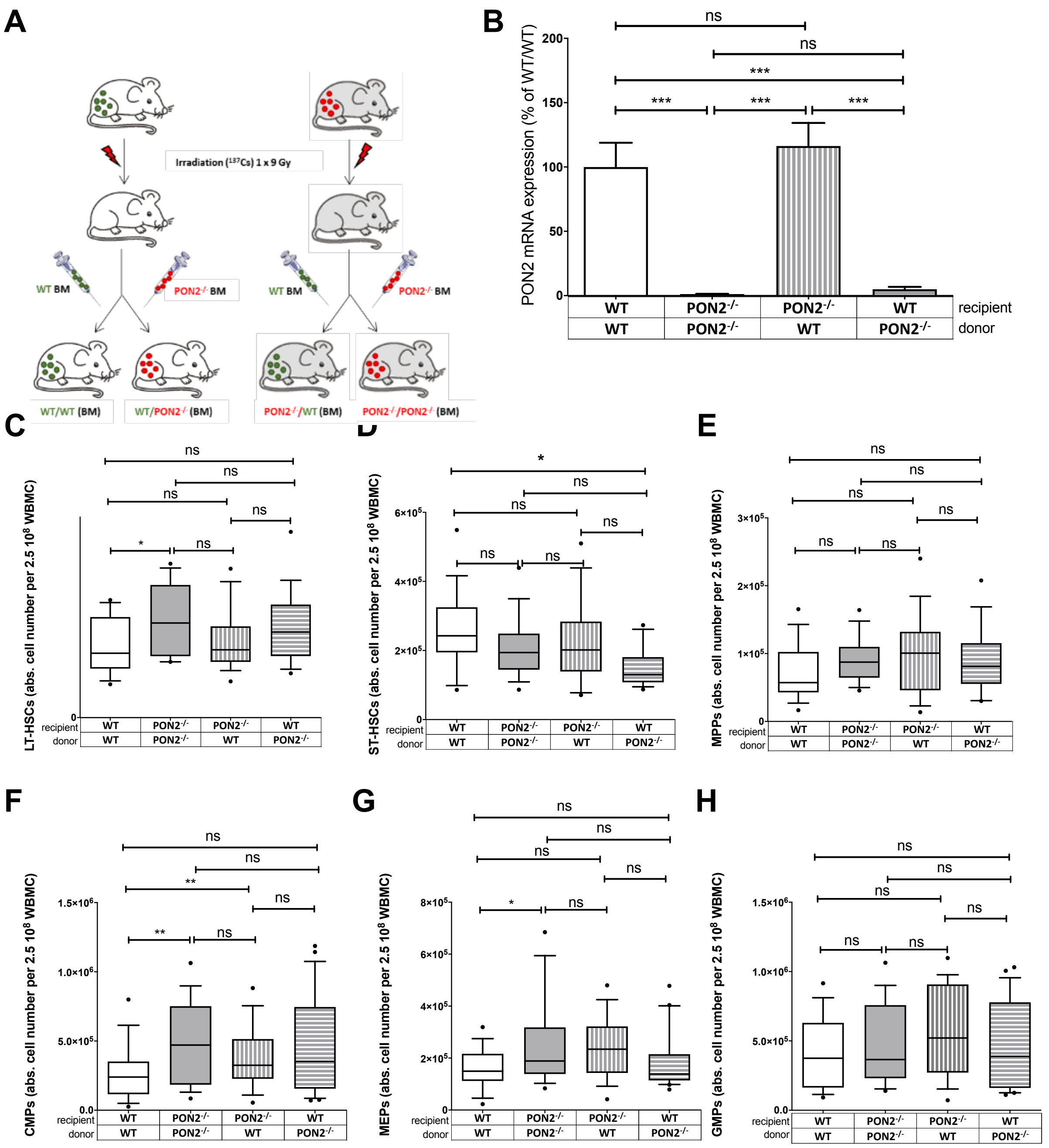

Figure S2. In young $Pon2^{-/-}$ mice, reciprocal BM transplantation reveals cell intrinsic as well as extrinsic phenotypes. (A) Experimental scheme for reciprocal transplantation. (B) Verification of engraftment of hematopoietic stem cells and reconstitution of hematopoiesis by donor stem cells in recipient mice using qRT-PCR based analysis of *Pon2* mRNA expression in isolated blood cells of transplanted animals (n=8-10). Results are presented as percentage of WT/WT and normalized to *Gapd*- and *Actb-mRNA expression*. (C) Percentage of LT-, (D) ST-HSCs, (E) MPPs, (F) CMPs, (G) MEPs and (H) GMPs in WBMCs of BM-chimeras and transplantation controls (n=10-13). Box and whiskers; Whiskers: 10-90 percentile. * $p \leq 0.05$, ** $p \leq 0.01$, ns not significant; One-way Anova with Tukey's multiple comparisons test.

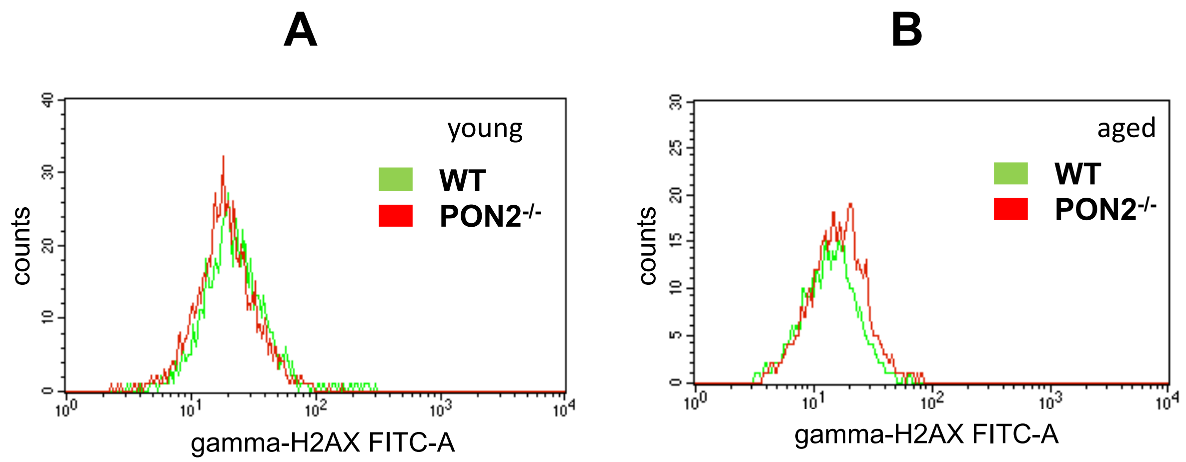

Figure S3. ***Pon2*$^{-/-}$ BMCs show no enhanced amount of DNA double-stand breaks in LSK cells.** Overlay of histograms showing the amount of DNA double-strand breaks in WT (green) and *Pon2*$^{-/-}$ (red) LSK cells, determined by anti-gamma-H2AX staining using BMCs from (A) young or (B) aged animals. One representative of 4 experiments is shown.

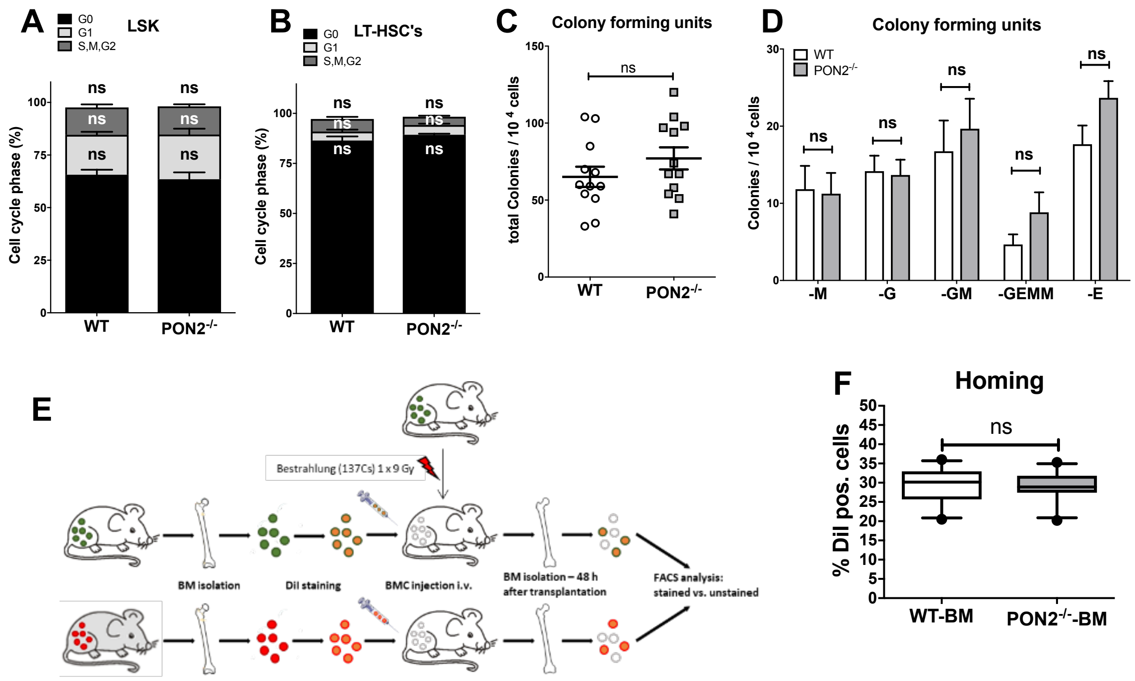

Figure S4. Bone marrow cells of young *Pon2*$^{-/-}$ and WT mice show no differences in cell cycle status, colony forming ability and homing. (A/B) Cell-cycle analysis of LSK (A) and LT-HSCs (B) of young WT and *Pon2*$^{-/-}$ mice as percentage within each cell-cycle phase (n=6). (C) Colony-forming unit assay (CFU): Total numbers of counted colonies and (D) observed CFU-M (colony-forming unit macrophage), CFU-G (colony-forming unit granulocyte), CFU-GM (colony-forming unit granulocyte/macrophage), CFU-GEMM (colony-forming unit granulocyte/ erythroid/macrophage/megakaryocyte) and CFU-E (colony-forming unit erythroid) colonies from 3x10^4 WT and *Pon2*$^{-/-}$ BMC's, 10-12 days after plating (n=12). (E) Experimental scheme of homing experiment. (F) Percentage of DiI positive WT and *Pon2*$^{-/-}$ BMCs isolated by flushing tibial and femoral bones of lethally irradiated WT recipient mice 48 hours after transplantation (n=10); ns not significant vs. WT, two tailed unpaired t-test.

A

LSK-fraction:

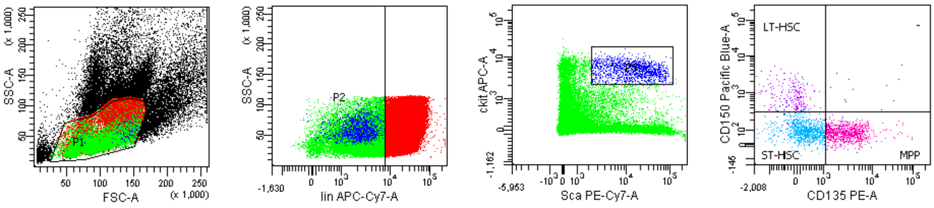

LT-HSCs: lin-, Sca1+, ckit+, CD150+, CD135-

ST-HSCs: lin-, Sca1+, ckit+, CD150-, CD135-

MPPs: lin-, Sca1+, ckit+, CD150-, CD135+

B

Oligopotent progenitors – myeloid lineage

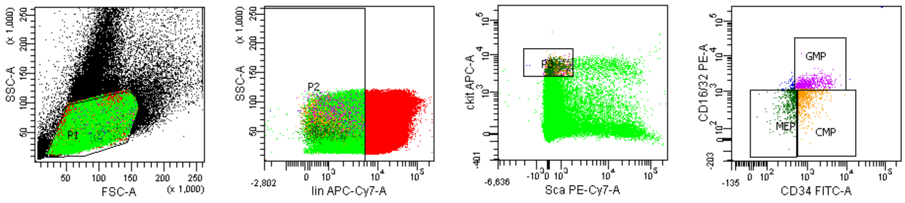

CMPs: lin-, Sca1-, ckit+, CD16/32-, CD34+

GMPs: lin-, Sca1-, ckit+, CD16/32+, CD34+

MEPs: lin-, Sca1-, ckit+, CD16/32-, CD34-

C

Apoptosis

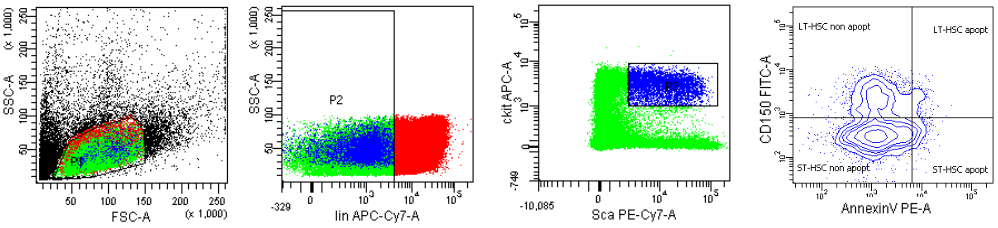

D

Competitive transplantation

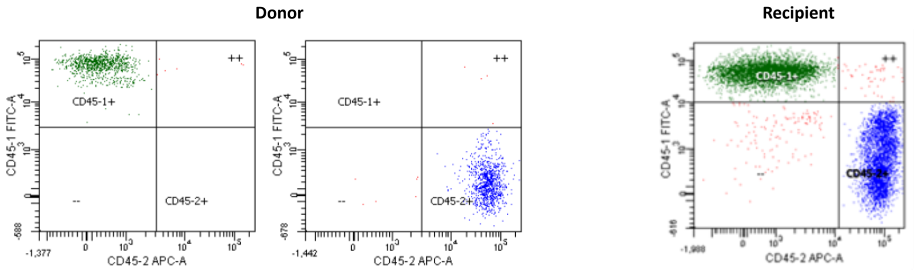

E

Cell cycle

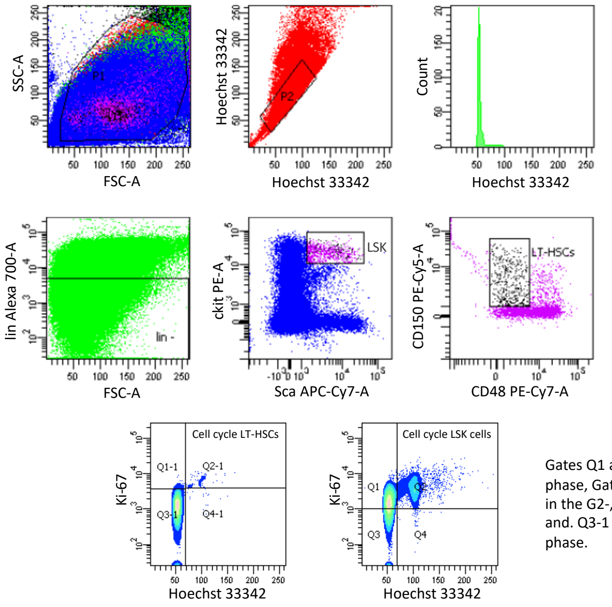

Gates Q1 and Q 1-1 represent cells in G1-phase, Gates Q2 and Q2-1 represent cells in the G2-, S- & M-phases and Gates Q3 and. Q3-1 represent cells Zellen the G0-phase.

F

Homing

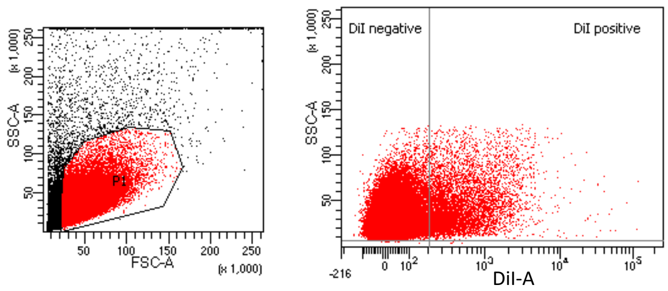

G **Total ROS-Level**

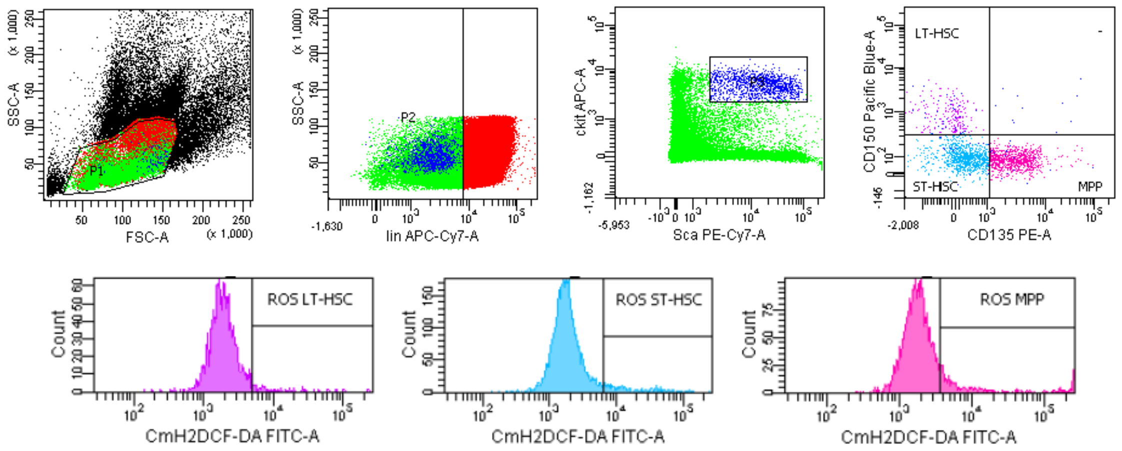

H **DNA-damage**

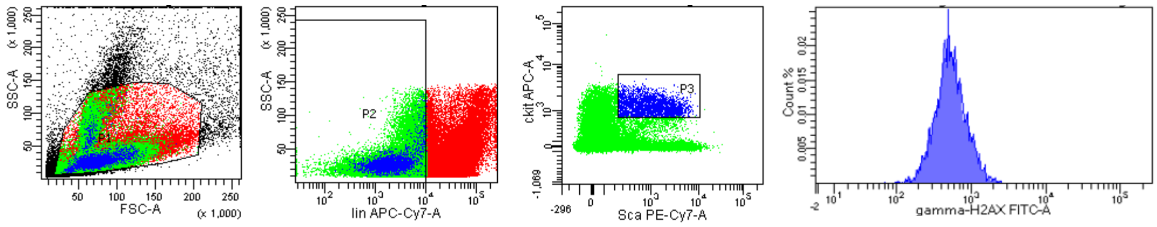

I **Erythrocyte Precursors**

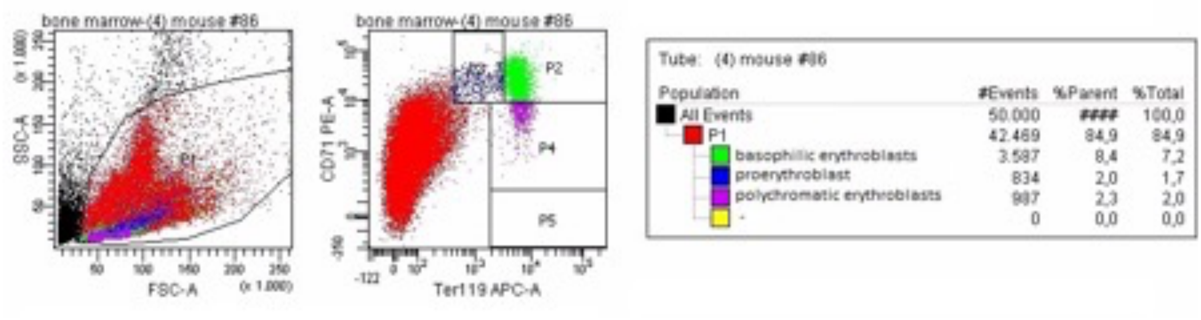

Figure S5. Gating strategies. Gating strategy for the measurement of **(A)** HSPC subpopulations within the LSK fraction of BM, **(B)** Oligopotent progenitors in the BM **(C)** apoptotic rate of LT- and ST-HSCs, **(D)** CD45.1 or CD45.2 cells in competitive transplantation experiments, **(E)** LSK cells and LT-HSCs in different stages of the cell cycle, **(F)** Dil-positive cells in homing experiments, **(G)** total ROS-Level of LT-, ST-HSCs and MPP, **(H)** DNA-damage experiments (**I**) Erythrocyte Precursor cells.

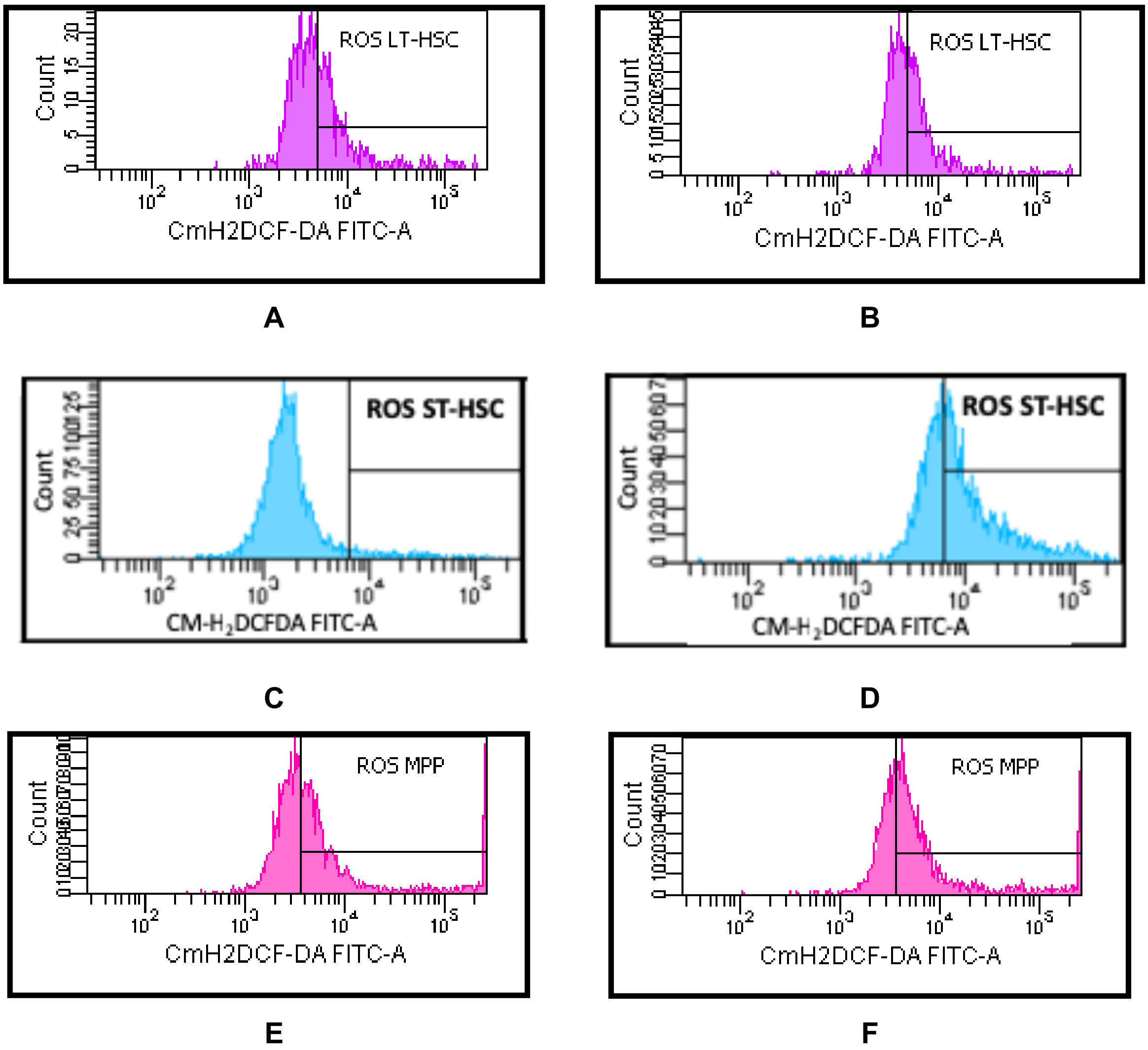

Figure S6. Representative histograms showing the DCF-DA data of LT- and ST-HSC as well as MPP isolated from young WT and Pon2$^{-/-}$ mice.

Total ROS-level in LT- (A/B), ST-HSCs (C/D) and MPPs (E/F) of young (2-3 months) WT (A/C/E) and *Pon2*$^{-/-}$ (B/D/E) mice stained with cell specific markers (LT-HSCs: Lin-, Sca1+, ckit+, CD135-, CD150+; ST-HSCs: Lin-, Sca1+, ckit+, CD135-, CD150-; MPPs: Lin-, Sca1+, ckit+, CD135+, CD150-) and H_2DCF-DA, analyzed by FACS (n=12-13).

Supplemental tables

Gene	forward (5' → 3')	reverse (5' → 3')	*probe* (FAM-5' → 3'-TAM)
Actb	AGAGGGAAATCGTGCG TGAC	CAATAGTGATGACCTGG CCGT	CACTGCCGCATCCTCTTCCT CCC
Gapdh	TCACCACCATGGAGAA GGC	GCTAAGCAGTTGGTGGT GCA	ATGCCCCCATGTTTGTGATG GGTGT
Pon2	CGGTATGTATGGGAAG ATGCTGAC	TTGTTGTTGCTGCTTCTG GGG	CCAATGGCCTGGCTTTCTTT

Table S1. Sequences of primers and probes used for qRT-PCR-based quantification of *Pon2* mRNA expression.

Gene name	Gene description	log2FoldChange	padj
**1110025M09Rik**	RIKEN cDNA 1110025M09 gene	-22.92	4.16E-12
**1110046J04Rik**	RIKEN cDNA 1110046J04 gene	-22.87	4.76E-12
**1700012C14Rik**	RIKEN cDNA 1700012C14 gene	-7.82	3.96E-03
**1700028J19Rik**	RIKEN cDNA 1700028J19 gene	22.66	6.59E-12
**1700088E04Rik**	RIKEN cDNA 1700088E04 gene	-7.89	2.29E-02
**2410003L11Rik**	RIKEN cDNA 2410003L11 gene	-8.65	4.50E-02
**2600006K01Rik**	RIKEN cDNA 2600006K01 gene	-22.45	9.93E-12
**2610300M13Rik**	RIKEN cDNA 2610300M13 gene	-24.05	4.24E-13
**2610507I01Rik**	RIKEN cDNA 2610507I01 gene	-4.26	1.89E-02
**3010001F23Rik**	RIKEN cDNA 3010001F23 gene	-22.86	4.76E-12

**3110045C21Rik**	RIKEN cDNA 3110045C21 gene	-8.79	3.91E-02
**3110067C02Rik**	RIKEN cDNA 3110067C02 gene	23.02	3.41E-12
**3632454L22Rik**	RIKEN cDNA 3632454L22 gene	-6.93	1.11E-02
**4930431P19Rik**	RIKEN cDNA 4930431P19 gene	-7.23	2.52E-02
**4930438A08Rik**	RIKEN cDNA 4930438A08 gene	-24.04	6.70E-16
**4930525G20Rik**	RIKEN cDNA 4930525G20 gene	21.50	7.02E-11
**4930594C11Rik**	RIKEN cDNA 4930594C11 gene	8.05	4.97E-02
**4933415A04Rik**	RIKEN cDNA 4933415A04 gene	8.55	8.61E-03
**4933431G14Rik**	RIKEN cDNA 4933431G14 gene	-23.84	6.36E-13
**5430434F05Rik**	RIKEN cDNA 5430434F05 gene	-7.42	4.92E-06
**6430548M08Rik**	RIKEN cDNA 6430548M08 gene	-6.82	4.68E-03
**6820402A03Rik**	RIKEN cDNA 6820402A03 gene	-10.01	7.11E-07
**9130208D14Rik**	RIKEN cDNA 9130208D14 gene	7.27	7.40E-06
**AA414768**	expressed sequence AA414768	20.65	5.90E-10
**Aatk**	apoptosis-associated tyrosine kinase	-23.43	1.02E-14
**Abca17**	ATP-binding cassette. sub-family A (ABC1). member 17	22.41	1.06E-11
**Abcc2**	ATP-binding cassette. sub-family C (CFTR/MRP). member 2	9.31	2.12E-03
**AC157931.1**	#WERT!	21.64	5.17E-11

**Adgre1**	adhesion G protein-coupled receptor E1	-22.92	4.16E-12
**Adtrp**	androgen dependent TFPI regulating protein	-23.21	2.40E-12
**Ajuba**	ajuba LIM protein	-9.70	9.36E-03
**Amdhd1**	amidohydrolase domain containing 1	-23.11	2.33E-14
**Aqr**	aquarius	-1.09	3.71E-02
**Arhgap22**	Rho GTPase activating protein 22	21.88	3.53E-11
**Arntl2**	aryl hydrocarbon receptor nuclear translocator-like 2	-24.19	3.01E-13
**Atp6v0e2**	ATPase. H+ transporting. lysosomal V0 subunit E2	8.41	2.98E-10
**Atp7b**	ATPase. Cu++ transporting. beta polypeptide	23.38	3.30E-14
**Atxn7l1os1**	ataxin 7-like 1. opposite strand 1	-23.23	2.35E-12
**AU019990**	expressed sequence AU019990	-7.94	1.59E-02
**B3galnt1**	UDP-GalNAc:betaGlcNAc beta 1.3-galactosaminyltransferase. polypeptide 1	-24.41	1.77E-13
**BC018473**	cDNA sequence BC018473	16.36	4.59E-76
**BC067074**	cDNA sequence BC067074	-7.78	3.64E-02
**Blk**	B lymphoid kinase	23.63	1.87E-13
**C230066G23Rik**	RIKEN cDNA C230066G23 gene	21.40	6.51E-11
**C78859**	expressed sequence C78859	-23.49	1.33E-12
**Catsperg1**	cation channel sperm associated auxiliary subunit gamma 1	-8.28	1.10E-02
**Ccdc136**	coiled-coil domain containing 136	22.68	6.30E-12
**Ccdc155**	coiled-coil domain containing 155	8.89	1.22E-02

**Ccdc62**	coiled-coil domain containing 62	-2.83	3.87E-03
**Ccnb1ip1**	cyclin B1 interacting protein 1	8.84	1.27E-02
**Cd19**	CD19 antigen	23.92	5.72E-13
**Cd276**	CD276 antigen	23.15	2.67E-12
**Cd300c2**	CD300C molecule 2	-24.03	4.33E-13
**Cd4**	CD4 antigen	21.76	4.59E-11
**Cd83**	CD83 antigen	-24.37	2.51E-17
**Cdk5rap1**	CDK5 regulatory subunit associated protein 1	3.20	6.62E-07
**Cdyl**	chromodomain protein. Y chromosome-like	-3.30	3.06E-02
**Chst7**	carbohydrate (N-acetylglucosamino) sulfotransferase 7	-8.01	2.70E-02
**Cpt1c**	carnitine palmitoyltransferase 1c	-7.15	4.97E-02
**Crisp2**	cysteine-rich secretory protein 2	-22.79	5.34E-12
**Crnde**	colorectal neoplasia differentially expressed (non-protein coding)	8.71	4.48E-02
**Csrp1**	cysteine and glycine-rich protein 1	2.75	1.02E-02
**Csrp3**	cysteine and glycine-rich protein 3	8.70	4.19E-02
**Ctse**	cathepsin E	3.88	3.43E-11
**Ctsg**	cathepsin G	-25.25	1.01E-15
**Cxcl3**	chemokine (C-X-C motif) ligand 3	-23.38	1.65E-12
**Cxcr4**	chemokine (C-X-C motif) receptor 4	2.10	3.17E-03
**Dagla**	diacylglycerol lipase. alpha	-23.51	1.28E-12
**Dcbld1**	discoidin. CUB and LCCL domain containing 1	-8.65	4.80E-02
**Depdc7**	DEP domain containing 7	8.93	3.61E-02
**Depp1**	DEPP1 autophagy regulator	9.45	1.46E-02

**Deptor**	DEP domain containing MTOR-interacting protein	1.08	1.49E-02
**Dleu7**	deleted in lymphocytic leukemia. 7	22.29	1.40E-11
**Dok7**	docking protein 7	-22.63	6.85E-12
**Efhd1**	EF hand domain containing 1	22.04	2.50E-11
**Eif5al3-ps**	eukaryotic translation initiation factor 5A-like 3. pseudogene	2.92	2.60E-05
**Entpd3**	ectonucleoside triphosphate diphosphohydrolase 3	-23.45	2.33E-14
**Ephb4**	Eph receptor B4	-8.51	3.47E-03
**Eps8**	epidermal growth factor receptor pathway substrate 8	-4.81	3.35E-02
**F2**	coagulation factor II	22.60	7.32E-12
**F730016J06Rik**	RIKEN cDNA F730016J06 gene	-22.81	5.34E-12
**Fam171b**	family with sequence similarity 171. member B	-9.20	2.93E-03
**Fam198a**	family with sequence similarity 198. member A	8.36	2.93E-02
**Fau-ps2**	Finkel-Biskis-Reilly murine sarcoma virus (FBR-MuSV) ubiquitously expressed (fox derived). pseudogene 2	-8.39	2.33E-03
**Fbxl12os**	F-box and leucine-rich repeat protein 12. opposite strand	7.70	1.45E-03
**Fbxo2**	F-box protein 2	22.32	1.31E-11
**Fbxo27**	F-box protein 27	-9.76	3.63E-04
**Fmn1**	formin 1	7.96	1.64E-02
**Fndc11**	fibronectin type III domain containing 11	23.48	1.33E-12
**Ftl2-ps**	ferritin light polypeptide 2. pseudogene	9.65	2.71E-04
**Gal3st3**	galactose-3-O-sulfotransferase 3	8.52	8.35E-03

**Gdpd5**	glycerophosphodiester phosphodiesterase domain containing 5	-24.10	1.37E-15
**Ggps1**	geranylgeranyl diphosphate synthase 1	-1.03	9.70E-03
**Gimap7**	GTPase. IMAP family member 7	-9.99	1.94E-04
**Glrp1**	glutamine repeat protein 1	-8.33	3.53E-03
**Gm10640**	predicted gene 10640	-23.55	1.16E-12
**Gm10704**	predicted pseudogene 10704	6.05	1.31E-11
**Gm11645**	predicted gene 11645	5.36	1.19E-04
**Gm11772**	predicted gene 11772	6.97	1.20E-02
**Gm11963**	predicted gene 11963	8.28	1.16E-02
**Gm12312**	predicted gene 12312	6.26	1.65E-06
**Gm12663**	predicted gene 12663	5.39	3.11E-02
**Gm12758**	predicted gene 12758	-6.75	3.11E-02
**Gm13262**	predicted gene 13262	8.87	3.90E-02
**Gm13571**	predicted gene 13571	21.88	3.48E-11
**Gm13594**	predicted gene 13594	-6.38	3.77E-02
**Gm14094**	predicted gene 14094	6.15	4.43E-05
**Gm15916**	predicted gene 15916	-22.99	3.61E-12
**Gm15989**	predicted gene 15989	-22.46	8.16E-12
**Gm16045**	predicted gene 16045	-7.05	4.40E-02
**Gm16059**	predicted gene 16059	7.37	4.80E-02
**Gm16083**	predicted gene 16083	-9.40	1.50E-05
**Gm16157**	predicted gene 16157	22.69	6.20E-12
**Gm16185**	predicted gene 16185	-7.82	1.60E-02
**Gm16523**	predicted gene. 16523	-8.60	2.32E-04
**Gm16754**	predicted gene. 16754	-8.32	9.77E-05
**Gm17088**	predicted gene 17088	-7.25	3.21E-02
**Gm17098**	predicted gene 17098	-22.14	1.97E-11
**Gm18860**	predicted gene. 18860	7.79	7.53E-11
**Gm1966**	predicted gene 1966	-4.33	3.55E-17
**Gm20658**	predicted gene 20658	-22.93	4.16E-12
**Gm26672**	predicted gene. 26672	-7.30	2.43E-02
**Gm26719**	predicted gene. 26719	-23.46	1.38E-12

**Gm26797**	predicted gene. 26797	8.84	4.07E-02
**Gm26814**	predicted gene. 26814	6.68	7.27E-03
**Gm26862**	predicted gene. 26862	8.03	2.23E-06
**Gm26865**	predicted gene. 26865	9.33	1.68E-03
**Gm26896**	predicted gene. 26896	-8.65	2.91E-08
**Gm27184**	predicted gene 27184	-8.36	9.85E-03
**Gm28370**	predicted gene 28370	10.68	1.56E-04
**Gm28438**	predicted gene 28438	5.64	6.81E-19
**Gm28707**	predicted gene 28707	8.61	2.38E-04
**Gm29590**	predicted gene 29590	-22.66	6.59E-12
**Gm2974**	predicted gene 2974	5.03	9.39E-03
**Gm31728**	predicted gene. 31728	-7.28	3.52E-02
**Gm35082**	predicted gene. 35082	9.87	2.14E-06
**Gm36963**	predicted gene. 36963	6.64	4.39E-02
**Gm37010**	predicted gene. 37010	9.57	5.09E-03
**Gm37053**	predicted gene. 37053	22.82	7.60E-14
**Gm37120**	predicted gene. 37120	-9.02	9.39E-03
**Gm37176**	predicted gene. 37176	9.13	4.73E-02
**Gm37366**	predicted gene. 37366	-22.62	7.07E-12
**Gm37415**	predicted gene. 37415	21.75	4.69E-11
**Gm37482**	predicted gene. 37482	-22.70	6.20E-12
**Gm37520**	predicted gene. 37520	-8.55	3.67E-05
**Gm37521**	predicted gene. 37521	23.41	1.54E-12
**Gm37566**	predicted gene. 37566	7.51	1.53E-02
**Gm37706**	predicted gene. 37706	-10.01	1.68E-04
**Gm37802**	predicted gene. 37802	-23.13	2.73E-12
**Gm38042**	predicted gene. 38042	-24.20	1.23E-16
**Gm38075**	predicted gene. 38075	8.65	2.49E-04
**Gm38140**	predicted gene. 38140	23.28	2.04E-12
**Gm38156**	predicted gene. 38156	-23.08	3.03E-12
**Gm38375**	predicted gene. 38375	-22.24	1.04E-11
**Gm38376**	predicted gene. 38376	-8.62	1.77E-02
**Gm38843**	predicted gene. 38843	22.24	1.58E-11
**Gm39307**	predicted gene. 39307	8.53	2.86E-02
**Gm44044**	predicted gene. 44044	-8.69	2.05E-02
**Gm44075**	predicted gene. 44075	7.42	2.47E-03

**Gm44086**	predicted gene. 44086	21.93	3.19E-11
**Gm44152**	predicted gene. 44152	9.49	7.36E-05
**Gm44571**	predicted gene 44571	-22.49	9.29E-12
**Gm44676**	predicted gene 44676	-5.61	4.26E-02
**Gm45159**	predicted gene 45159	-22.72	6.14E-12
**Gm45191**	predicted gene 45191	23.73	2.70E-16
**Gm45217**	predicted gene 45217	22.19	1.74E-11
**Gm45220**	predicted gene 45220	-9.59	2.43E-04
**Gm45240**	predicted gene 45240	-22.60	7.32E-12
**Gm45693**	predicted gene 45693	-22.24	6.59E-12
**Gm45854**	predicted gene 45854	-8.81	4.97E-03
**Gm4587**	predicted gene 4587	4.83	2.48E-03
**Gm47283**	predicted gene. 47283	-3.55	1.49E-02
**Gm48604**	predicted gene. 48604	22.43	1.02E-11
**Gm48714**	predicted gene. 48714	-22.38	1.16E-11
**Gm5292**	predicted gene 5292	8.20	1.52E-18
**Gm5464**	predicted gene 5464	-23.80	7.02E-13
**Gm5483**	predicted gene 5483	24.20	3.12E-18
**Gm6123**	predicted gene 6123	4.06	2.95E-04
**Gm6225**	predicted gene 6225	-7.10	1.52E-02
**Gm7224**	predicted gene 7224	6.29	7.27E-03
**Gm7639**	predicted gene 7639	6.46	7.23E-05
**Gm7889**	predicted gene 7889	5.42	2.63E-02
**Gm7890**	predicted gene 7890	-6.24	4.78E-02
**Gm8420**	predicted gene 8420	9.31	1.39E-81
**Gm8979**	predicted gene 8979	5.57	4.13E-03
**Gm9903**	predicted gene 9903	-9.46	1.46E-02
**Gnb4**	guanine nucleotide binding protein (G protein). beta 4	1.72	9.25E-06
**Gnpda2**	glucosamine-6-phosphate deaminase 2	-12.61	6.55E-35
**Gp1bb**	glycoprotein Ib. beta polypeptide	-23.14	2.68E-12
**Gpr84**	G protein-coupled receptor 84	-24.27	2.49E-13
**Gulo**	gulonolactone (L-) oxidase	-23.58	1.10E-12

**Gvin1**	GTPase. very large interferon inducible 1	-8.24	2.19E-06
**H2-T3**	histocompatibility 2. T region locus 3	-8.82	2.93E-02
**Hbb-bt**	hemoglobin. beta adult t chain	-7.07	6.25E-07
**Heatr5b**	HEAT repeat containing 5B	2.09	5.10E-03
**Hist1h4m**	histone cluster 1. H4m	-7.81	3.70E-06
**Hist3h2ba**	histone cluster 3. H2ba	-7.89	2.32E-03
**Hormad2**	HORMA domain containing 2	22.71	6.14E-12
**Hoxb6**	homeobox B6	-5.20	1.89E-02
**Htatip2**	HIV-1 Tat interactive protein 2	-11.86	1.04E-25
**Id3**	inhibitor of DNA binding 3	22.79	5.34E-12
**Ifi204**	interferon activated gene 204	6.65	2.11E-04
**Ifi208**	interferon activated gene 208	21.64	4.29E-11
**Ifi209**	interferon activated gene 209	-24.63	1.00E-13
**Ifi27**	interferon. alpha-inducible protein 27	1.08	2.82E-02
**Igf2bp2**	insulin-like growth factor 2 mRNA binding protein 2	-3.60	2.62E-02
**Ighv6-3**	immunoglobulin heavy variable 6-3	8.69	4.32E-02
**Igsf9**	immunoglobulin superfamily. member 9	-9.30	1.99E-03
**Il22ra2**	interleukin 22 receptor. alpha 2	23.61	1.06E-12
**Insr**	insulin receptor	-1.34	3.32E-02
**Irs3**	insulin receptor substrate 3	-23.91	5.73E-13
**Itln1**	intelectin 1 (galactofuranose binding)	21.94	3.11E-11
**Junos**	jun proto-oncogene. opposite strand	21.48	6.42E-11
**Kbtbd12**	kelch repeat and BTB (POZ) domain containing 12	-6.59	4.24E-02
**Klc3**	kinesin light chain 3	8.76	3.97E-02
**Klrb1f**	killer cell lectin-like receptor subfamily B member 1F	-22.72	6.14E-12

**Lancl1**	LanC (bacterial lantibiotic synthetase component C)-like 1	-1.21	1.28E-03
**Ldb2**	LIM domain binding 2	8.05	3.42E-02
**Lipc**	lipase. hepatic	-23.48	1.63E-14
**Lmln**	leishmanolysin-like (metallopeptidase M8 family)	-7.37	9.01E-03
**Lmntd2**	lamin tail domain containing 2	9.57	1.09E-02
**Loxl1**	lysyl oxidase-like 1	20.94	3.02E-10
**Mcf2**	mcf.2 transforming sequence	9.38	4.07E-02
**Meg3**	maternally expressed 3	-7.45	2.43E-04
**Met**	met proto-oncogene	7.16	4.07E-02
**Mkrn2**	makorin. ring finger protein. 2	1.41	4.32E-02
**Mmp25**	matrix metallopeptidase 25	-24.63	1.00E-13
**Mrpl48-ps**	mitochondrial ribosomal protein L48 pseudogene	4.46	3.29E-05
**mt-Nd3**	mitochondrially encoded NADH dehydrogenase 3	-9.54	2.02E-11
**mt-Tf**	mitochondrially encoded tRNA phenylalanine	3.22	4.01E-02
**mt-Tr**	mitochondrially encoded tRNA arginine	-4.89	3.03E-02
**Mtmr9**	myotubularin related protein 9	3.83	5.56E-06
**Mycn**	v-myc avian myelocytomatosis viral related oncogene. neuroblastoma derived	-1.04	1.63E-02
**Myo1b**	myosin IB	-8.90	3.43E-02
**Mysm1**	myb-like. SWIRM and MPN domains 1	1.01	4.16E-02
**Myzap**	myocardial zonula adherens protein	21.43	7.47E-11
**Nhsl2**	NHS-like 2	-6.32	2.25E-02
**Nlrp5-ps**	NLR family. pyrin domain containing 5. pseudogene	-22.59	7.36E-12
**Nphp4**	nephronophthisis 4 (juvenile) homolog (human)	-9.08	6.79E-04

**Nudt10**	nudix (nucleoside diphosphate linked moiety X)-type motif 10	21.49	7.53E-11
**Olfml2b**	olfactomedin-like 2B	-22.75	5.98E-12
**Olfr56**	olfactory receptor 56	5.22	3.33E-02
**Olfr65**	olfactory receptor 65	-9.88	2.60E-03
**Olfr658**	olfactory receptor 658	-9.38	4.28E-02
**Omd**	osteomodulin	-8.58	1.85E-02
**Oosp1**	oocyte secreted protein 1	-22.72	6.14E-12
**Opn3**	opsin 3	-23.89	5.92E-13
**P2ry13**	purinergic receptor P2Y. G-protein coupled 13	23.20	3.55E-17
**Pcdhgb7**	protocadherin gamma subfamily B. 7	-23.04	3.28E-12
**Pfn2**	profilin 2	-24.48	1.15E-17
**Pfn4**	profilin family. member 4	-22.66	6.60E-12
**Pgf**	placental growth factor	21.59	4.91E-11
**Phf24**	PHD finger protein 24	-23.76	7.54E-13
**Pih1d2**	PIH1 domain containing 2	-23.61	1.07E-12
**Plp1**	proteolipid protein (myelin) 1	8.97	2.05E-02
**Pmel**	premelanosome protein	-23.61	2.33E-14
**Polm**	polymerase (DNA directed). mu	-25.19	1.59E-15
**Pon2**	paraoxonase 2	-2.13	3.03E-03
**Pou6f1**	POU domain. class 6. transcription factor 1	8.05	1.89E-02
**Ppox**	protoporphyrinogen oxidase	-1.69	1.52E-02
**Ppp1r3e**	protein phosphatase 1. regulatory subunit 3E	-9.39	2.85E-04
**Prom1**	prominin 1	-9.11	2.66E-02
**Prox2os**	prospero homeobox 2 opposite strand	-7.14	4.32E-02
**Prss8**	protease. serine 8 (prostasin)	9.15	5.67E-03
**Psmb5**	proteasome (prosome. macropain) subunit. beta type 5	-1.05	5.27E-05

**Ptdss1**	phosphatidylserine synthase 1	1.35	2.94E-03
**Ptgfrn**	prostaglandin F2 receptor negative regulator	-23.38	1.63E-14
**Pycard**	PYD and CARD domain containing	-1.06	8.15E-03
**Pycr1**	pyrroline-5-carboxylate reductase 1	-9.46	1.42E-02
**Qrsl1**	glutaminyl-tRNA synthase (glutamine-hydrolyzing)-like 1	2.04	7.27E-03
**Rab36**	RAB36. member RAS oncogene family	24.89	3.57E-22
**Rasl11a**	RAS-like. family 11. member A	-25.25	3.79E-21
**Rbm45**	RNA binding motif protein 45	3.69	1.91E-07
**Rdh5**	retinol dehydrogenase 5	7.86	1.68E-03
**Recql4**	RecQ protein-like 4	7.75	1.80E-03
**Rem2**	rad and gem related GTP binding protein 2	8.23	8.49E-03
**Rgs9**	regulator of G-protein signaling 9	23.87	5.96E-13
**Rhbg**	Rhesus blood group-associated B glycoprotein	-8.11	1.34E-02
**Rhov**	ras homolog family member V	22.45	9.93E-12
**Rora**	RAR-related orphan receptor alpha	-1.30	3.86E-03
**Rprm**	reprimo. TP53 dependent G2 arrest mediator candidate	-22.99	3.61E-12
**Rps13-ps1**	ribosomal protein S13. pseudogene 1	7.55	2.87E-168
**Rps27a-ps1**	ribosomal protein S27A. pseudogene 1	-6.79	2.62E-02
**Sbsn**	suprabasin	9.09	2.82E-02
**Sdcbp2**	syndecan binding protein (syntenin) 2	23.43	1.49E-12
**Sema6a**	sema domain. transmembrane domain (TM).	22.79	5.34E-12

	and cytoplasmic domain. (semaphorin) 6A		
**Serpinb10**	serine (or cysteine) peptidase inhibitor. clade B (ovalbumin). member 10	9.55	1.32E-05
**Serpine2**	serine (or cysteine) peptidase inhibitor. clade E. member 2	-7.37	2.95E-02
**Serping1**	serine (or cysteine) peptidase inhibitor. clade G. member 1	-23.30	2.03E-12
**Sh2d4a**	SH2 domain containing 4A	-24.00	5.74E-16
**Sh2d6**	SH2 domain containing 6	-23.59	1.09E-12
**Shisa7**	shisa family member 7	-22.26	1.16E-11
**Sla**	src-like adaptor	1.12	4.64E-02
**Slc12a4**	solute carrier family 12. member 4	-9.53	1.99E-03
**Slc16a5**	solute carrier family 16 (monocarboxylic acid transporters). member 5	-7.54	4.50E-03
**Slc23a4**	solute carrier family 23 member 4	-24.45	1.88E-17
**Slc6a12**	solute carrier family 6 (neurotransmitter transporter. betaine/GABA). member 12	-22.79	5.34E-12
**Slc6a9**	solute carrier family 6 (neurotransmitter transporter. glycine). member 9	7.38	3.39E-02
**Slc7a8**	solute carrier family 7 (cationic amino acid transporter. y+ system). member 8	2.89	2.58E-03
**Slc9a9**	solute carrier family 9 (sodium/hydrogen exchanger). member 9	-9.62	9.85E-03
**Smim6**	small integral membrane protein 6	-2.37	3.57E-02
**Smkr-ps**	smal lysine rich protein 1. pseudogene	21.96	3.00E-11
**Smtnl2**	smoothelin-like 2	-23.63	1.87E-14

**Snord32a**	small nucleolar RNA. C/D box 32A	4.66	3.28E-02
**Sox6**	SRY (sex determining region Y)-box 6	1.49	3.32E-03
**Spaca9**	sperm acrosome associated 9	-8.05	3.92E-02
**Spata18**	spermatogenesis associated 18	-9.43	1.33E-03
**Spint1**	serine protease inhibitor. Kunitz type 1	-22.70	6.20E-12
**St6galnac1**	ST6 (alpha-N-acetyl-neuraminyl-2.3-beta-galactosyl-1.3)-N-acetylgalactosaminide alpha-2.6-sialyltransferase 1	8.29	1.13E-02
**St6galnac2**	ST6 (alpha-N-acetyl-neuraminyl-2.3-beta-galactosyl-1.3)-N-acetylgalactosaminide alpha-2.6-sialyltransferase 2	6.09	7.42E-03
**Stfa2l1**	stefin A2 like 1	23.71	8.34E-13
**Ston2**	stonin 2	-9.82	1.64E-06
**Stx1a**	syntaxin 1A (brain)	-22.19	9.93E-12
**Tarm1**	T cell-interacting. activating receptor on myeloid cells 1	21.73	4.91E-11
**Tcte2**	t-complex-associated testis expressed 2	8.85	3.64E-02
**Tent5c**	terminal nucleotidyltransferase 5C	23.17	2.57E-12
**Tert**	telomerase reverse transcriptase	-9.50	9.80E-05
**Tgm1**	transglutaminase 1. K polypeptide	-10.27	8.06E-07
**Them6**	thioesterase superfamily member 6	7.98	1.77E-02
**Tmem136**	transmembrane protein 136	-7.46	1.75E-03
**Tmem17**	transmembrane protein 17	11.24	3.60E-13
**Tmem171**	transmembrane protein 171	-22.57	7.69E-12

**Tmem268**	transmembrane protein 268	6.15	4.98E-02
**Tmem44**	transmembrane protein 44	8.67	3.48E-03
**Tmem52b**	transmembrane protein 52B	22.82	5.21E-12
**Tnfsf13os**	tumor necrosis factor (ligand) superfamily. member 13. opposite strand	-3.96	2.47E-03
**Tnfsf4**	tumor necrosis factor (ligand) superfamily. member 4	-9.28	1.89E-02
**Trim12a**	tripartite motif-containing 12A	-10.04	1.68E-47
**Trim34b**	tripartite motif-containing 34B	8.94	5.18E-08
**Trim5**	tripartite motif-containing 5	-3.88	1.74E-07
**Trp53inp2**	transformation related protein 53 inducible nuclear protein 2	-9.67	1.78E-07
**Trpc7**	transient receptor potential cation channel. subfamily C. member 7	-23.14	2.68E-12
**Ugt1a8**	UDP glucuronosyltransferase 1 family. polypeptide A8	6.38	2.15E-04
**Vmn1r90**	vomeronasal 1 receptor 90	-23.15	2.67E-12
**Vstm5**	V-set and transmembrane domain containing 5	23.02	1.63E-14
**Wdfy1**	WD repeat and FYVE domain containing 1	2.04	5.62E-03
**Wdr54**	WD repeat domain 54	-7.64	4.19E-02
**Wdr86**	WD repeat domain 86	23.12	2.73E-12
**Wfdc18**	WAP four-disulfide core domain 18	21.97	2.92E-11
**Zfp322a**	zinc finger protein 322A	-1.78	1.38E-02
**Zfp960**	zinc finger protein 960	4.92	2.17E-03

Table S2. Differential expressed genes identified using DESeq2 and whole genome RNAseq data from HSCs isolated from WT and *Pon2*$^{-/-}$ animals.

Shown are the gene names. gene descriptions. the Log2-fold change in gene expression and the adjusted p-value. Genes discussed in the text are marked in red.

pathway	source	external_id	genes	p-value	q-value
**CXCR4-mediated signaling events**	PID	cxcr4_pathway	CD4; GNAZ; RGS1; BLK; CXCR4	1.62E-04	2.01E-02
**hiv-1 defeats host-mediated resistance by cem15**	BioCarta	vifpathway	CD4; CXCR4	3.43E-04	2.01E-02
**Binding and entry of HIV virion**	Reactome	R-HSA-173107	CD4; CXCR4	3.43E-04	2.01E-02
**Calcium Regulation in the Cardiac Cell**	Wiki-pathways	WP536	GNB4; GNAZ; RGS1; CAMK2B; RGS9	1.89E-03	5.26E-02
**Cooperation of PDCL (PhLP1) and TRiC/CCT in G-protein beta folding**	Reactome	R-HSA-6814122	GNB4; RGS9; GNAZ	2.08E-03	5.26E-02
**activation of csk by camp-dependent protein kinase inhibits signaling through the t cell receptor**	BioCarta	cskpathway	CD4; F2; CXCR4	2.37E-03	5.26E-02
**G alpha (i) signalling events**	Reactome	R-HSA-418594	GNB4; RDH5; GNAZ; P2RY13; RGS1;	2.44E-03	5.26E-02

			CXCR4; GPR18; RGS9		
**effects of calcineurin in keratinocyte differentiation**	BioCarta	calcineurinpathway	SP3; CAMK2B	2.59E-03	5.26E-02
**ion channels and their functional role in vascular endothelium**	BioCarta	raccpathway	F2; CAMK2B; CXCR4	2.69E-03	5.26E-02
**Early Phase of HIV Life Cycle**	Reactome	R-HSA-162594	CD4; CXCR4	3.46E-03	6.09E-02
**Codeine and Morphine Metabolism**	Wikipathways	WP1604	UGT1A8; ABCC2	3.94E-03	6.31E-02
**Semaphorin interactions**	Reactome	R-HSA-373755	MET; CDK5R1; SEMA6A	6.43E-03	9.42E-02
**Photodynamic therapy-induced NFE2L2 (NRF2) survival signaling**	Wikipathways	WP3612	NFE2L2; ABCC2	8.09E-03	9.42E-02
**Visual signal transduction: Cones**	PID	cone_pathway	RDH5; RGS9	8.09E-03	9.42E-02
**Visual signal transduction: Rods**	PID	rhodopsin_pathway	RDH5; RGS9	8.79E-03	9.42E-02
**regulation of ck1/cdk5 by type 1**	BioCarta	ck1pathway	CDK5R1; CAMK2B	8.79E-03	9.42E-02

**glutamate receptors**					
**Rho GTPase cycle**	Reactome	R-HSA-194840	MCF2; ARHGAP22; RHOV; DEPDC7	9.52E-03	9.42E-02

Table S3. Pathways enriched in HSCs isolated from *Pon2*$^{-/-}$ animals.

Shown are the pathway names, the source, the external ID, the gene names, the p-value and the q-value.

pathway	source	external_id	genes	p-value	q-value
**Intrinsic Pathway of Fibrin Clot Formation**	Reactome	R-HSA-140837	SERPING1; SERPINE2; GP1BB	3,67E-04	4,59E-02
**Activation of Matrix Metalloproteinases**	Reactome	R-HSA-1592389	MMP25; MMP15; CTSG	1,03E-03	5,77E-02
**Neurotoxicity of clostridium toxins**	Reactome	R-HSA-168799	STX1A; SV2A	1,80E-03	5,77E-02
**Formation of Fibrin Clot (Clotting Cascade)**	Reactome	R-HSA-140877	SERPING1; SERPINE2; GP1BB	2,01E-03	5,77E-02
**Salmonella infection - Homo sapiens (human)**	KEGG	path:hsa05132	PYCARD; PFN2; PFN4; CXCL3	2,31E-03	5,77E-02
**Metabolism of amino acids and derivatives**	Reactome	R-HSA-71291	RPS13; SAT1; RPL15; RPL39L; AMDHD1; SLC6A12; PYCR1	6,29E-03	1,17E-01
**GABA synthesis, release, reuptake and degradation**	Reactome	R-HSA-888590	STX1A; SLC6A12	6,58E-03	1,17E-01

Table S4. Pathways decreased in HSCs isolated from *Pon2*$^{-/-}$ animals.

Shown are the pathway names, the source, the external ID, the gene names, the p-value and the q-value.
